# UNC-41/Stonin Functions with AP2 to Recycle Synaptic Vesicles in *Caenorhabditis elegans*

**DOI:** 10.1371/journal.pone.0040095

**Published:** 2012-07-10

**Authors:** Gregory P. Mullen, Kiely M. Grundahl, Mingyu Gu, Shigeki Watanabe, Robert J. Hobson, John A. Crowell, John R. McManus, Eleanor A. Mathews, Erik M. Jorgensen, James B. Rand

**Affiliations:** 1 Genetic Models of Disease Research Program, Oklahoma Medical Research Foundation, Oklahoma City, Oklahoma, United States of America; 2 Howard Hughes Medical Institute and Department of Biology, University of Utah, Salt Lake City, Utah, United States of America; Brown University, United States of America

## Abstract

The recycling of synaptic vesicles requires the recovery of vesicle proteins and membrane. Members of the stonin protein family (*Drosophila* Stoned B, mammalian stonin 2) have been shown to link the synaptic vesicle protein synaptotagmin to the endocytic machinery. Here we characterize the *unc-41* gene, which encodes the stonin ortholog in the nematode *Caenorhabditis elegans*. Transgenic expression of *Drosophila stonedB* rescues *unc-41* mutant phenotypes, demonstrating that UNC-41 is a *bona fide* member of the stonin family. In *unc-41* mutants, synaptotagmin is present in axons, but is mislocalized and diffuse. In contrast, UNC-41 is localized normally in synaptotagmin mutants, demonstrating a unidirectional relationship for localization. The phenotype of *snt-1 unc-41* double mutants is stronger than *snt-1* mutants, suggesting that UNC-41 may have additional, synaptotagmin-independent functions. We also show that *unc-41* mutants have defects in synaptic vesicle membrane endocytosis, including a ∼50% reduction of vesicles in both acetylcholine and GABA motor neurons. These endocytic defects are similar to those observed in *apm-2* mutants, which lack the µ2 subunit of the AP2 adaptor complex. However, no further reduction in synaptic vesicles was observed in *unc-41 apm-2* double mutants, suggesting that UNC-41 acts in the same endocytic pathway as µ2 adaptin.

## Introduction

The release of neurotransmitters at synapses occurs through the regulated fusion of synaptic vesicles with the plasma membrane. The recovery of synaptic vesicle membrane and proteins through endocytosis is dependent on a large complex of proteins associated with clathrin [1]. Membrane is recruited by the AP2 clathrin adaptor complex, which binds PIP_2_ (phosphatidylinositol 4,5-bisphosphate) in the plasma membrane. The AP2 complex also recruits synaptic vesicle proteins to endocytic sites [2]. Some synaptic vesicle proteins are recognized by the presence of a dileucine motif that binds the σ2 subunit, or a YxxΦ motif that binds the µ2 subunit of AP2 [3]. In contrast, other synaptic vesicle proteins require specific adaptors to recruit them to endocytic sites. The adaptors CALM and AP180, for example, are required to recruit the v-SNARE synaptobrevin into recycling synaptic vesicles [4]–[8]. Similarly, UNC-46/BAD-LAMP is an adaptor for the vesicular GABA transporter (VGAT) in *C. elegans* [9]. Stonins are likely to be the adaptors that recruit the calcium sensor synaptotagmin to endocytosing synaptic vesicles [10].

The founding member of the stonin protein family, stoned B, was identified in *Drosophila* in genetic screens for temperature-sensitive paralytic mutants [11]. *Drosophila stoned* mutants exhibit behavioral, electrophysiological, and ultrastructural defects that indicate that synaptic vesicle recycling is severely compromised [12]. The *stoned* locus encodes two proteins, stoned A (STNA) and stoned B (STNB); STNA proteins are only found in insects, while proteins similar to STNB are found in all metazoans [13]. Two STNB homologs, stonin 1 and stonin 2, have been identified in mice and humans [14]. Of the two mammalian homologs, stonin 2 has a greater similarity to STNB, and like the *Drosophila* protein, has been implicated in recycling of synaptic vesicle proteins [15], [16].

Experiments in flies and mice suggest that stonins are linked to synaptotagmin function. STNB binds to the C2B domain of *Drosophila* synaptotagmin [17], [18], and stonin 2 is capable of binding either C2 domain of mammalian synaptotagmin, although it preferentially binds to C2A [19]. In both flies and mice, binding to synaptotagmin is mediated by the stonin μ-homology domain (µHD). *Drosophila stoned* mutants exhibit defects in synaptotagmin localization and synaptic vesicle recycling [12], and stonin 2 is required for endocytosis of synaptotagmin in mammalian cell culture [16], [19]. Intriguingly, it has been reported that overexpression of synaptotagmin in *Drosophila* rescues the lethality and synaptic vesicle recycling defects observed in *stoned* mutants [20]. Thus, a generally accepted model is that the major function of stonins is to recruit synaptotagmin to endocytic sites.

We now show that the absence of UNC-41/stonin in *C. elegans* leads to defects in synaptotagmin localization and synaptic vesicle endocytosis. The endocytic defects are similar to those observed in *apm-2* mutants, which lack the µ2 subunit of the AP2 adaptor complex. No further reduction in synaptic vesicles was observed in *unc-41 apm-2* double mutants, suggesting that UNC-41/stonin acts in the same endocytic pathway as µ2 adaptin.

## Results

### Cloning and Genomic Organization of the *unc-41* Gene

Mutations in the *C. elegans unc-41* gene result in uncoordinated movement, resistance to inhibitors of cholinesterase, slow growth, and small adult size; these phenotypes are usually associated with defects in acetylcholine (ACh) release [21]–[24]. In addition, *unc-41* animals display a defecation defect associated with loss of γ-aminobutyric acid (GABA) function [25], [26]. These phenotypes suggest that *unc-41* encodes a protein important for the release of most or all neurotransmitter types. In addition, *unc-41* mutants resemble synaptotagmin-deficient (*snt-1*) mutants [27], suggesting that the *unc-41* gene products play a role in synaptic vesicle fusion or recycling.

**Figure 1:**
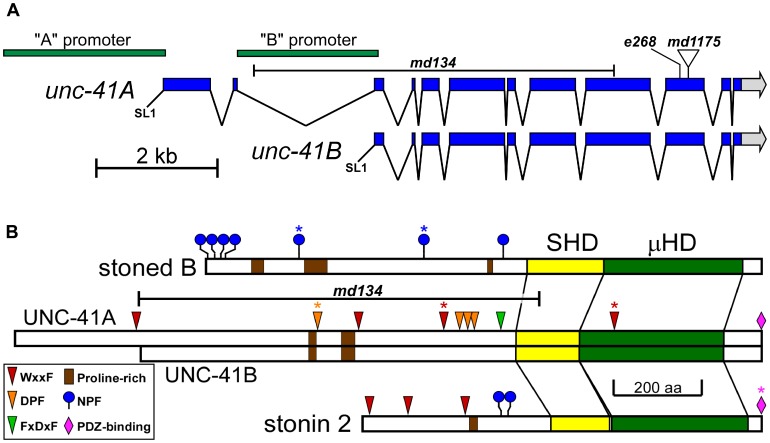
The *unc-41* gene and UNC-41 proteins. (A) The *unc-41* gene consists of 12 exons spanning ∼9 kb of genomic DNA. The two promoter regions are shown in green, and several *unc-41* mutations are indicated. (B) The *unc-41* gene products are members of the stonin family. Shown are features of the UNC-41A (∼188 kDa) and B (∼160 kDa) proteins, as well as the *Drosophila* stoned B and human stonin 2 proteins. Each of these proteins possesses a central stonin-homology domain (SHD) and a C-terminal µ-homology domain (µHD); significant sequence similarity among the proteins is limited to these domains. The brown rectangles indicate proline-rich domains (defined as a sequence of ≥21 amino acids containing ≥33% prolines). Blue circles indicate NPF motifs. Three motifs, shown as triangles, interact with the α-ear domains of the AP2 complex: red triangles indicate WxxF motifs [29], [30], orange triangles indicate DPF motifs [28], and the green triangle indicates an FxDxF motif [28]. The pink diamonds indicate C-terminal PDZ domain-binding motifs [31]. Asterisks indicate that the marked site is not conserved even among closely related species.

We cloned *unc-41* by transposon tagging, and found that it corresponds to the predicted gene C27H6.1. We note that this sequence has also been designated “*apt-10*” (see Materials and Methods). A genomic clone containing the 9 kb coding region plus ∼3.5 kb of upstream sequence fully rescued the *unc-41* locomotion and defecation phenotypes (data not shown). The longest gene product, *unc-41A*, consists of 12 exons with a 5′ transpliced SL1 leader (Figure 1A). A shorter gene product, *unc-41B*, consists of ten exons, beginning with exon 3, and is also transpliced to the SL1 leader. Northern analysis revealed two low abundance transcripts (5.3 kb and 4.5 kb); the sizes and hybridization patterns of these transcripts are consistent with the structures of the two gene products shown in Figure 1A. We found no evidence for additional structural heterogeneity in the *unc-41* gene transcripts.

Sequence comparisons suggest that the *unc-41* gene encodes a homolog of *Drosophila* STNB and mouse stonin 2 (Figure 1B). Most prominent are the two signature stonin domains: a stonin homology domain (SHD) followed by a C-terminal µ homology domain (µHD). In these conserved regions, UNC-41 and STNB are ∼45% identical, and UNC-41 and mouse stonin 2 are ∼31% identical. Although the stonins do not show significant conservation in their N-terminal regions, these regions contain motifs which interact with clathrin ancillary proteins [10]. Unlike *Drosophila* and mammalian stonins, UNC-41 lacks the NPF motifs which bind the EH domains of eps15 and intersectin [10]. However, UNC-41 does contain DPF, FxDxF, and WxxF motifs, which potentially bind the ear domains of the α subunit of AP2 [10], [28]–[30], and a C-terminal ΦxΦ Type 2 PDZ-binding motif [31] capable of binding to many synaptic PDZ domain-containing proteins (Figure 1B). Like STNB and stonin 2, UNC-41 contains proline-rich regions predicted to bind SH3 domains such as those found in intersectin [13]. There is no apparent similarity between any part of the UNC-41 proteins and the *stonedA* gene product other than α AP2-binding DPF motifs.

### Analysis of *unc-41* Mutants

We identified the sequence alterations associated with 33 *unc-41* alleles (Table S1 and Figure S1). Sixteen of the mutations are associated with DNA rearrangements leading to altered fragment lengths. These include 13 alleles with Tc1 transposon insertions, a 176-bp tandem duplication, and two significant (≥100-bp) deletions. The remaining 17 alleles include six with base-substitutions and 11 with small insertions or deletions, all of which lead to termination codons or frameshifts. The canonical allele, *e268*, is associated with a base substitution in exon 10, which converts Trp1468 to a stop codon (Figures 1A and S1 and Table S1). It is noteworthy that we did not identify any missense mutations, even among the EMS-induced alleles.

We compared the swimming and pharyngeal pumping rates for animals homozygous for each of the alleles listed in Table S1 (except for the Tc1-insertion mutations), and all 19 strains examined had comparable behavioral deficits (swimming was 19.8±4.0% of wild type; pumping was 21.5±2.6% of wild type). This set of behavioral deficits represents the *unc-41* null phenotype, and all of these mutations appear to be null.

### *unc-41* Expression

**Figure 2:**
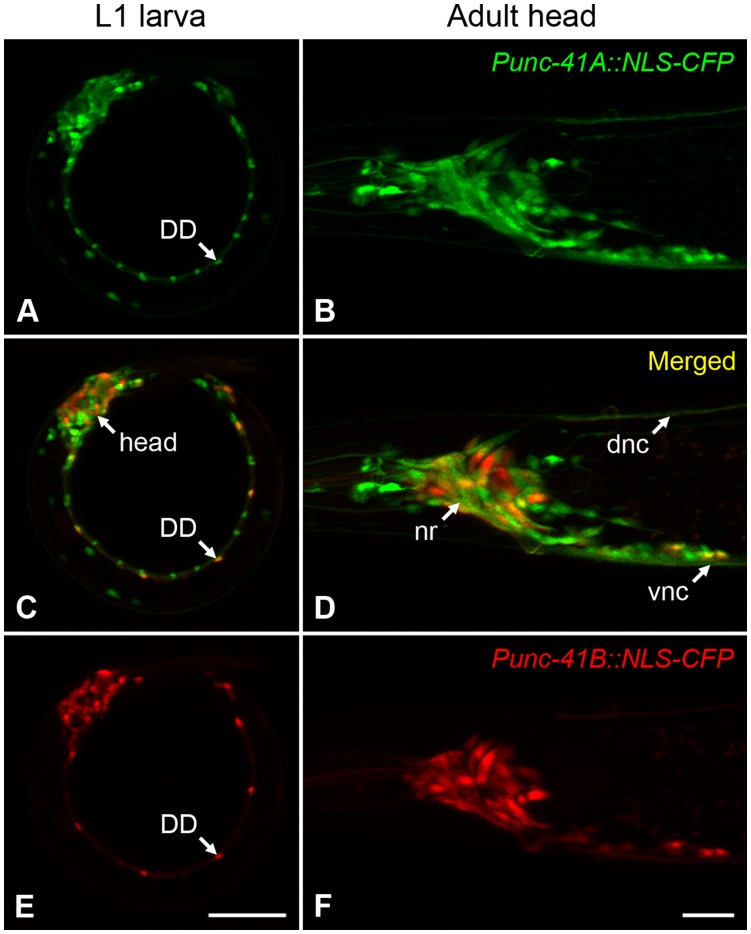
The *unc-41* gene products are differentially expressed in the *C. elegans* nervous system. Animals expressing the *Punc-41A::*NLS-CFP (green) and *Punc-41B::*NLS-YFP (red) transgenes were imaged on a confocal microscope. An L1 larva is shown in the left column (panels A, C, and E). One of the six GABA motor neurons (DD) is indicated. The animal’s head is in the upper left of the image, and the animal is positioned with its ventral surface on the inside of the arc (scale bar is 10 µm). Shown in the right column (panels B, D, and F) is the head region of an adult hermaphrodite. The nerve ring (nr), dorsal nerve cord (dnc), and ventral nerve cord (vnc) are indicated for reference. Anterior is to the left, ventral is down, and the bar is 10 µm.

We generated reporter constructs with the putative *unc-41A* regulatory region (2672-bp upstream of exon 1) or the *unc-41B* regulatory region (2343-bp upstream of exon 3) driving CFP or YFP. Expression of both reporters appeared to be restricted to the nervous system (Figure 2). The *unc-41A* reporter was expressed in most or all *C. elegans* neurons, including the GABA and ACh motor neurons, whereas the *unc-41B* reporter was expressed in a subset of neurons, including the GABA motor neurons (DD and VD cells) in the ventral nerve cord (Figure 2). A significant number of neurons did not express the *unc-41B* reporter; these include the ventral cord ACh motor neurons (VA, VB, DA, DB, VC, and AS cells). We note that expression of a single isoform (UNC-41B) from the *unc-41A* promoter rescues *unc-41* mutant phenotypes [19].

### UNC-41 is Localized to Synapses and Colocalizes with Synaptotagmin

**Figure 3:**
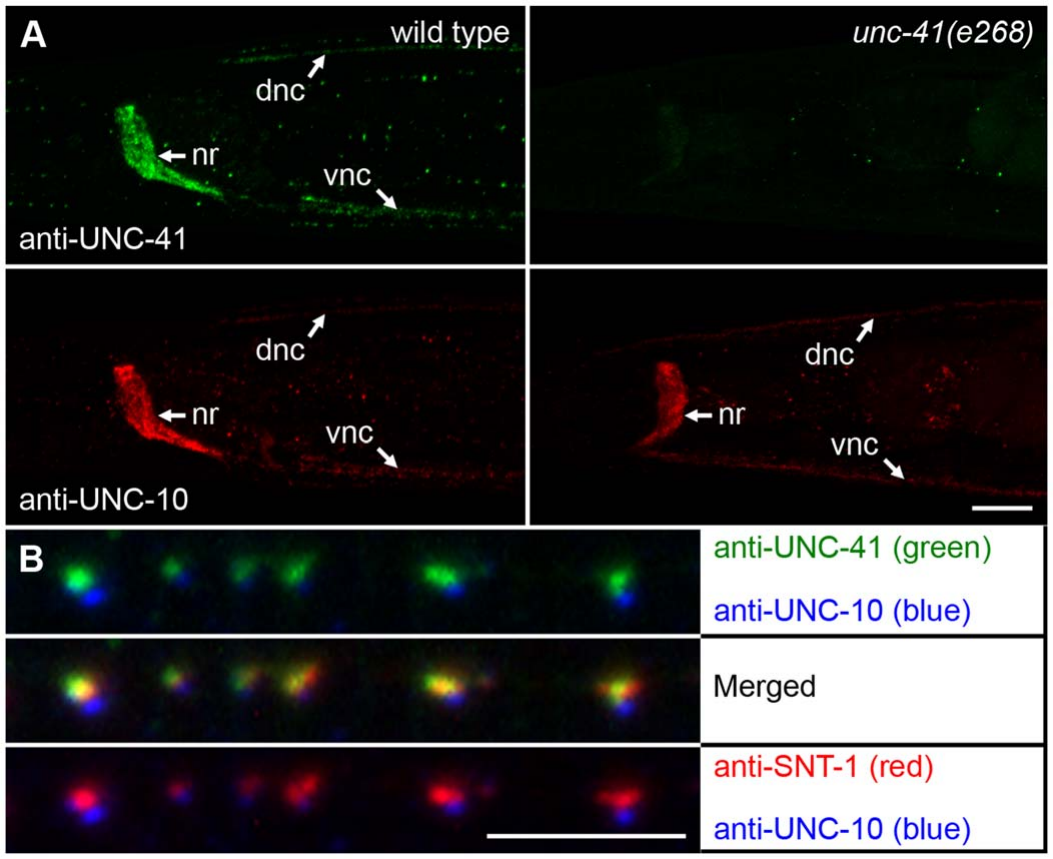
The UNC-41 proteins are localized to synaptic regions and colocalize with synaptic vesicle proteins. (A) Wild-type and *unc-41(e268)* animals were stained with antibodies to UNC-41 (green) and UNC-10/RIM (red). UNC-41 is specifically associated with synaptic regions: heavy punctate staining is observed in the nerve ring (nr), and the dorsal (dnc) and ventral (vnc) nerve cords. The UNC-41 proteins are absent in *unc-41(e268)* animals. Head region, anterior is to the left, ventral is down, and the bar is 10 µm. (B) Magnified view of anterior sublateral (SAB) nerve cords stained with antibodies to UNC-41 (green), synaptotagmin (SNT-1; red), and UNC-10/RIM (blue). UNC-41 staining overlaps with SNT-1, but does not colocalize significantly with UNC-10/RIM. Scale bar is 5 µm.

We raised polyclonal antibodies against a bacterially expressed UNC-41 fusion protein. This fusion protein includes ∼67 kDa from the C-terminal region, which is present in both UNC-41 isoforms (corresponding to the SHD and µHD). Anti-UNC-41 antibodies (gt216) were affinity-purified and used for immunofluorescence staining. We found that UNC-41 is specifically associated with synaptic regions in the *C. elegans* nervous system (Figure 3). Strong punctate staining was observed in the nerve ring and dorsal and ventral nerve cords, regions rich in synapses. Punctate staining was also associated with other nerve processes, including the sublateral nerve cords. In multiple-labeling experiments, UNC-41 was found to overlap significantly with synaptotagmin (Figure 3B). The distribution of endogenous UNC-41 was similar to that of a GFP::UNC-41 fusion protein (see below), and is consistent with observations of stonins in other organisms, including *Drosophila* and humans [15]. Staining was not observed in *unc-41(e268)* mutants (Figure 3A), demonstrating that the antibody is specific for UNC-41, and providing additional evidence that *e268* is a null allele.

### Synaptotagmin Localization Requires UNC-41

**Figure 4:**
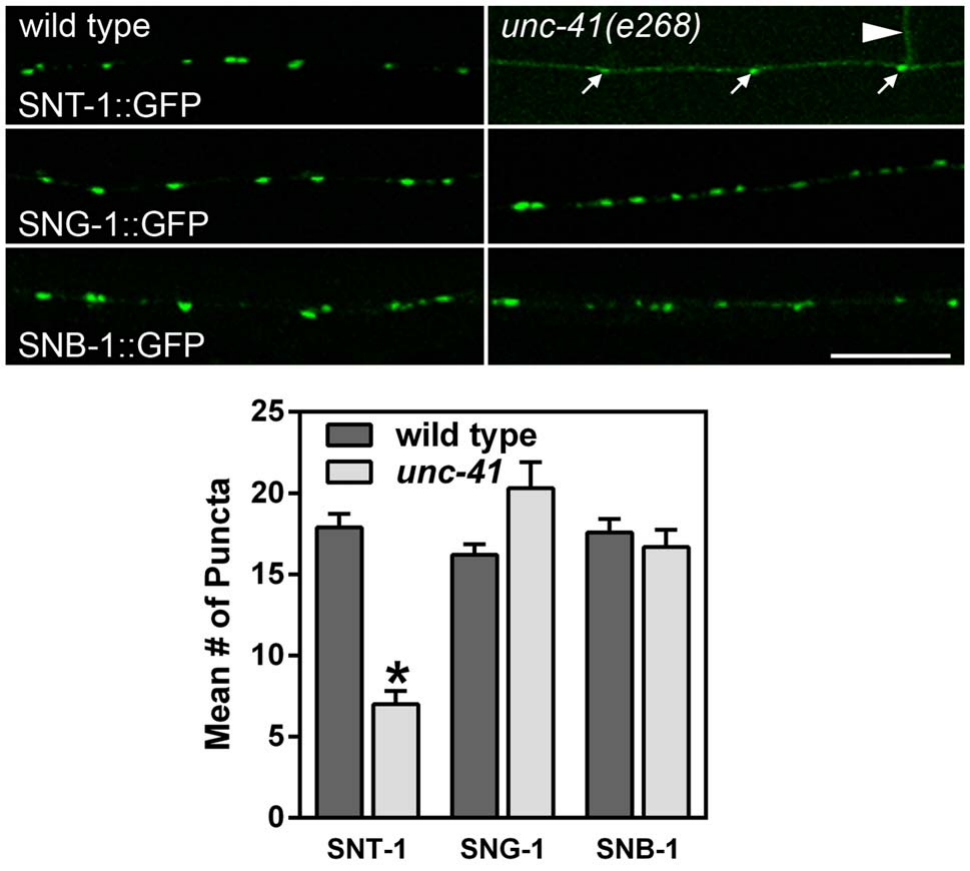
Upper: Localization of synaptotagmin to synapses is disrupted in *unc-41* mutants. Images are of body sublateral nerve cords approximately midway between the level of the retrovesicular ganglion and the vulva. In *unc-41* mutants, SNT-1::GFP is localized in fewer and smaller puncta (arrows), and is diffusely distributed in axons and commissures (arrowhead), whereas synaptobrevin and synaptogyrin have only minor localization defects. Scale bar is 10 µm. Lower: Quantification of puncta in sublateral nerve cords. The number of SNT-1::GFP, SNB-1::GFP, or SNG-1::GFP puncta were determined for wild type and *unc-41* mutants as described in Materials and Methods (*P<0.00001). Error bars indicate SEM.

In *Drosophila stoned* mutants, synaptotagmin is specifically reduced and mislocalized at presynaptic boutons [12]. We therefore examined the localization of synaptic vesicle proteins, including synaptotagmin (SNT-1), in the sublateral nerve cords of *unc-41* mutants. GFP-tagged vesicle proteins were expressed under control of their respective promoters; constructs were present as single copy insertions to avoid possible overexpression. Consistent with studies in *Drosophila* [12], tagged SNT-1 was reduced and mislocalized in *unc-41* mutants, and was observed in non-synaptic regions such as commissures (Figures 4 and S2). Although tagged SNT-1 was occasionally observed in puncta, such puncta were small compared to the wild type (Figure 4). Two other synaptic vesicle proteins, synaptobrevin and synaptogyrin, exhibited only minor localization defects (Figure 4). We quantified the puncta for each synaptic vesicle protein in the sublateral cords of mutant and wild type animals (Figure 4), and found that the number of synaptotagmin-containing puncta in *unc-41* mutants was significantly reduced compared with wild type, whereas synaptobrevin and synaptogyrin were near-normal. Furthermore, the trafficking and localization of two endocytosis-related proteins (UNC-57/endophilin and APA-2/α adaptin) were not affected by the absence of UNC-41 (Figure S3).

We extended these observations by examining the localization of GFP-tagged synaptic vesicle proteins in GABA motor neurons. As in the sublateral neurons, synaptotagmin was mislocalized along axons and was present in non-synaptic regions in *unc-41* mutants. These effects appear to be specific to synaptotagmin, because other synaptic vesicle proteins (Figure S3) as well as the endocytosis-related protein APM-2/μ2 adaptin [32], exhibited only mild defects in synaptic localization. Finally, antibody staining of synaptotagmin was dimmer and more diffuse in *unc-41* mutants, although once again, some synaptotagmin was still localized at synapses (data not shown). Together, these results suggest that UNC-41 is required to establish or maintain the localization of SNT-1 at synapses.

### UNC-41 Localization does not Require Synaptotagmin

**Figure 5:**
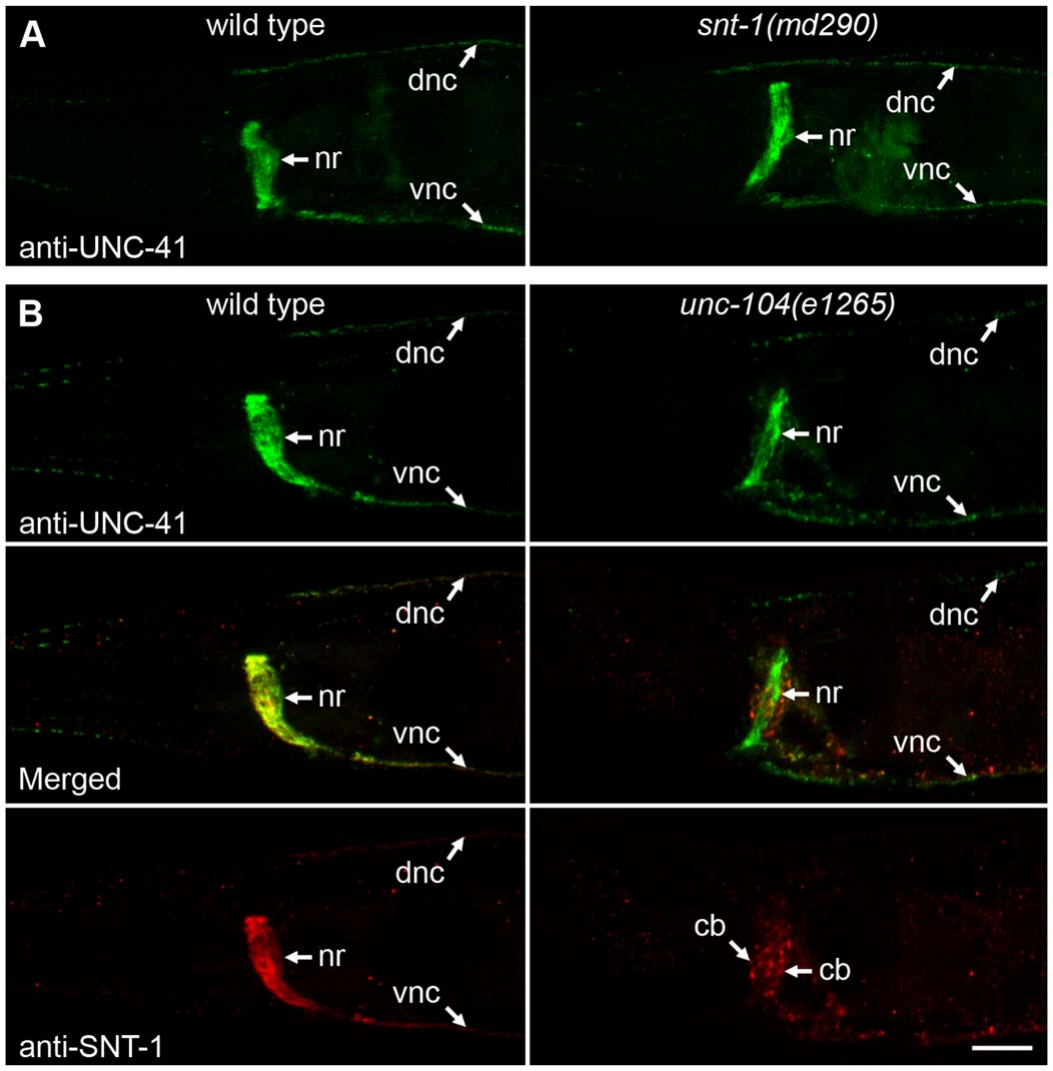
(A) Localization of UNC-41 is independent of synaptotagmin. Wild-type and *snt-1(md290)* young adult hermaphrodites were stained with antibodies to UNC-41. In both wild-type and mutant animals, UNC-41 is specifically associated with synaptic regions, including the nerve ring (nr) and dorsal (dnc) and ventral (vnc) nerve cords. (B) Synaptotagmin (SNT-1) but not UNC-41 requires the synaptic vesicle kinesin UNC-104 for transport to synapses. Wild-type and *unc-104(e1265)* animals were stained with antibodies to UNC-41 (green) and SNT-1 (red). Synaptotagmin is mislocalized to neuronal cell bodies (cb) and is no longer observed at synapses in the dorsal and ventral nerve cords in *unc-104(e1265),* whereas UNC-41 is still trafficked to synaptic regions. Slight accumulation of UNC-41 in neuronal cell bodies is also observed in *unc-104* mutants. All images are of head regions, anterior is to the left, ventral is down, and the scale bar is 10 µm.

In mammalian tissue culture cells, synaptotagmin recruits stonin 2 to the plasma membrane [19], suggesting that these two proteins are required reciprocally for localization at synapses. We therefore examined UNC-41 localization in *snt-1(md290)* mutants; *md290* is a null allele of *snt-1* [33]. We found that endogenous UNC-41, as assayed by immunostaining, was localized to synapses (Figure 5). We conclude that SNT-1 is not necessary for UNC-41 localization to synapses, which is consistent with results in *Drosophila* [20], [34].

Furthermore, it is unlikely that UNC-41 is localized to synapses by other synaptotagmin isoforms or synaptic vesicle proteins, even though stonin 2 also interacts with the synaptotagmin isoforms Syt2 and Syt9 at the cell surface [16]. In *C. elegans*, there are six synaptotagmin genes, *snt-1* to *snt-6*. We examined the localization of GFP::UNC-41B in two different synaptotagmin triple mutants: *snt-4 snt-1 snt-2* and *snt-6 snt-1 snt-3*, and observed that GFP::UNC-41B was localized to synapses in both of these triple mutants (Figure S4). To determine whether UNC-41 is localized to synapses by multiple synaptic vesicle proteins redundantly, we examined UNC-41 distribution in *unc-104* mutants; *unc-104* encodes a kinesin required for axonal transport of synaptic vesicle proteins [35], [36]. As expected, SNT-1 was strongly mislocalized to neuronal cell bodies in *unc-104* mutants (Figure 5). However, UNC-41 was still enriched in synaptic regions such as the nerve ring and nerve cords of *unc-104* mutants (Figure 5). These data suggest that synaptic localization of UNC-41 does not depend on SNT-1 or other *C. elegans* synaptotagmins, other synaptic vesicle components, or the synaptic vesicle kinesin UNC-104. We conclude that the relationship between UNC-41 and SNT-1 is non-reciprocal: UNC-41 localizes synaptotagmin to synapses, but synaptotagmin does not localize UNC-41.

### Heterologous Expression of STNB Rescues *unc-41* Mutant Phenotypes

**Figure 6:**
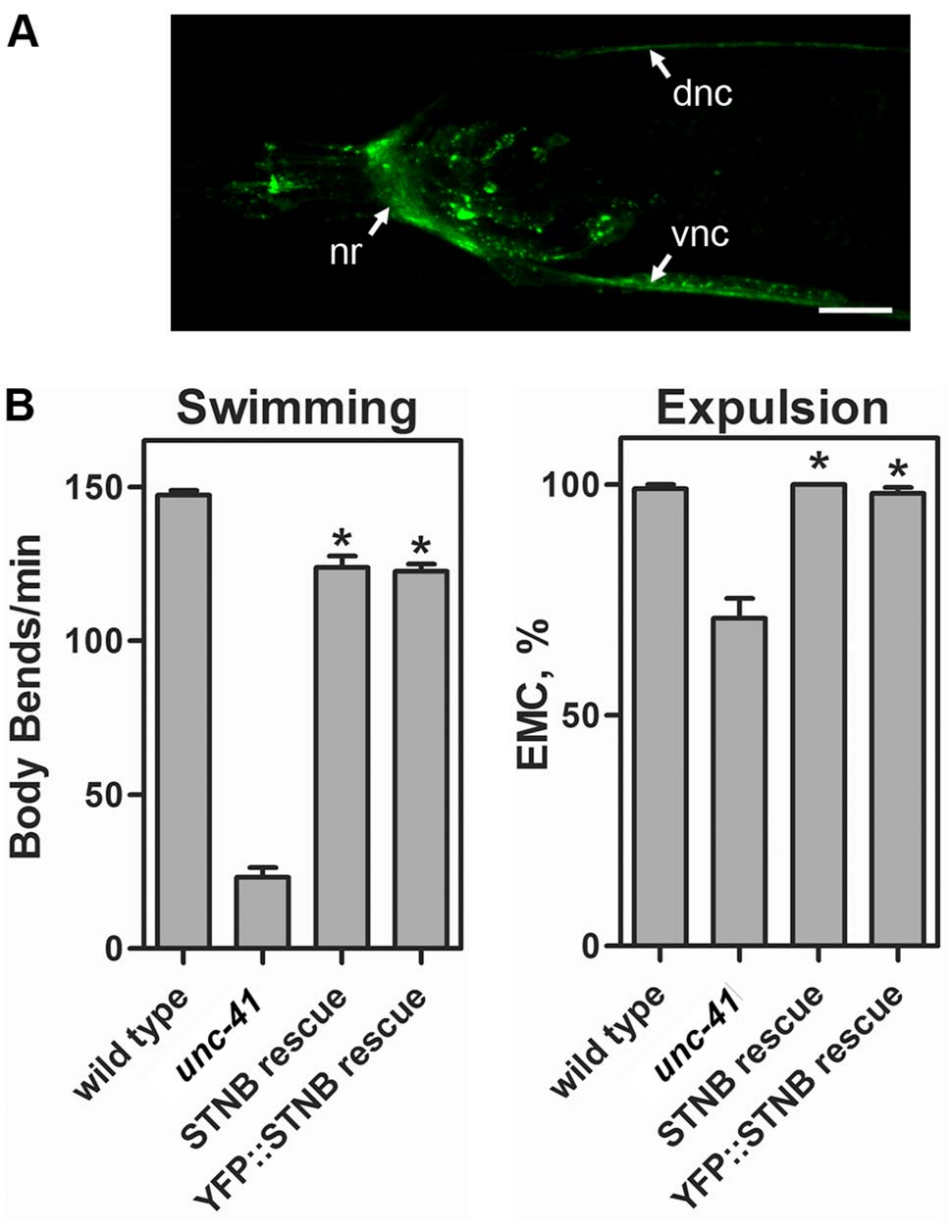
Phenotypic rescue of *unc-41* mutants by transgenic expression of *Drosophila* stoned B (STNB). (A) A YFP::STNB fusion protein is correctly trafficked and localized to synaptic regions. Shown is the head of a young adult hermaphrodite; anterior is to the left, and the scale bar is 10 µm. STNB is specifically associated with synaptic regions, including the nerve ring (nr) and dorsal (dnc) and ventral (vnc) nerve cords. (B) Behavioral analyses of young adult hermaphrodites expressing STNB or YFP::STNB transgenes. The strains analyzed were (from left to right) RM2086, RM2655, RM2683, and RM3644; genotypes are provided in List S1. Details of swimming and expulsion assays are in Materials and Methods. For swimming assays, each bar represents the mean number of body bends/min for 20 individuals of each strain. For expulsion assays, each bar represents the percentage of defecation cycles ending with an enteric muscle contraction (EMC, %) for 10 individuals of each strain. Error bars indicate SEM. Asterisks denote significant difference from *unc-41* (P<0.0001).

We expressed the *Drosophila* STNB protein, with or without an N-terminal YFP tag, under control of the *unc-41A* promoter. We found that the YFP::STNB protein was appropriately trafficked to synapses (Figure 6A), and that transgenic expression of STNB rescued *unc-41* mutant phenotypes (Figure 6B). We conclude that the UNC-41 and STNB proteins are functionally equivalent, despite the considerable sequence divergence of these molecules. We also observed that the YFP::STNB fusion protein was correctly localized to synaptic regions in *snt-1* mutants (data not shown); thus SNT-1 is not required for STNB localization.

### Analysis of *snt-1 unc-41* Double Mutants

**Figure 7:**
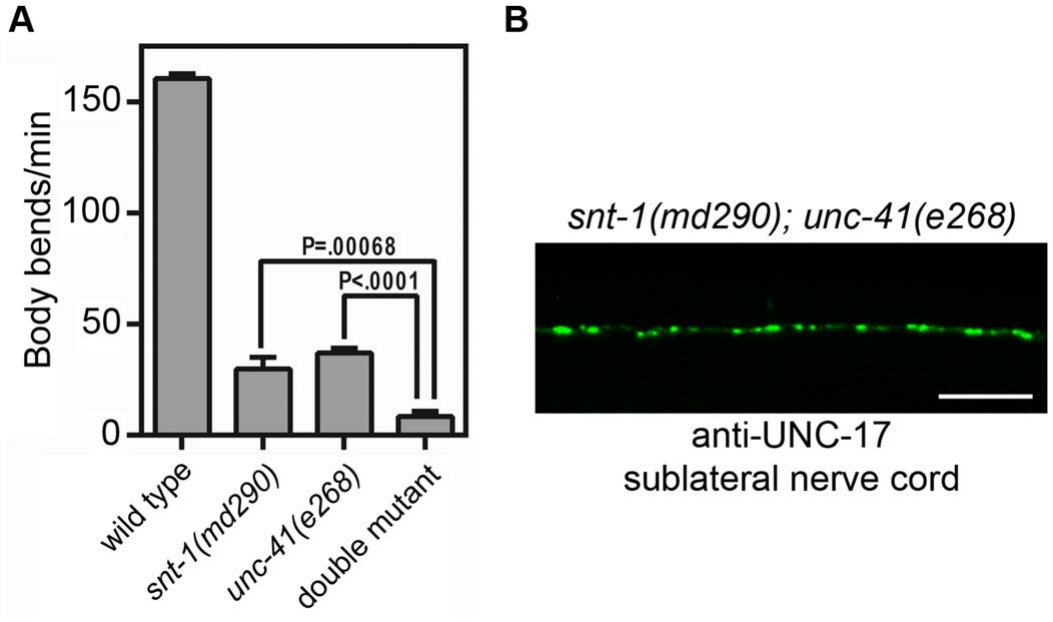
Analysis of *snt-1 unc-41* double mutants. (A) Swimming assays of wild type, *snt-1(md290)*, and *unc-41(e268)* single mutants, and *snt-1(md290); unc-41(e268)* double mutants (details in Materials and Methods). Each bar represents the mean number of body bends/min for 20 individuals of each strain; error bars indicate SEM. Comparable results were observed with *snt-1(md184); unc-41(e268)* and *snt-1(md290); unc-41(md152)* double mutants (data not shown). (B) Immunohistochemical staining for the synaptic vesicle protein UNC-17/VAChT in *snt-1(md290); unc-41(e268)* double mutants, demonstrating proper subcellular localization of this protein. Shown is a confocal image of cholinergic synapses in a body-sublateral nerve cord of a young adult hermaphrodite; anterior is to the left, and the scale bar is 10 µm.

Evaluation of double mutant phenotypes is a well-established genetic strategy to determine whether two genes function in the same pathway. We therefore constructed *snt-1 unc-41* double mutants using three different allelic combinations, and we observed that the swimming behavior of these double mutants was more impaired than either of the single mutants (Figure 7A). Thus, the SNT-1 and UNC-41 proteins do not appear to function exclusively in the same pathway. The localization of the synaptic vesicle proteins UNC-17/VAChT (Figure 7B) and synaptobrevin, as well as the active zone protein UNC-10/RIM (data not shown), were essentially normal in the double mutants. Thus, despite the absence of the SNT-1 and UNC-41 proteins, the presynaptic organization of cholinergic synapses appeared essentially normal.

### Overexpression of Synaptotagmin does not Rescue *unc-41* Mutant Phenotypes

**Figure 8:**
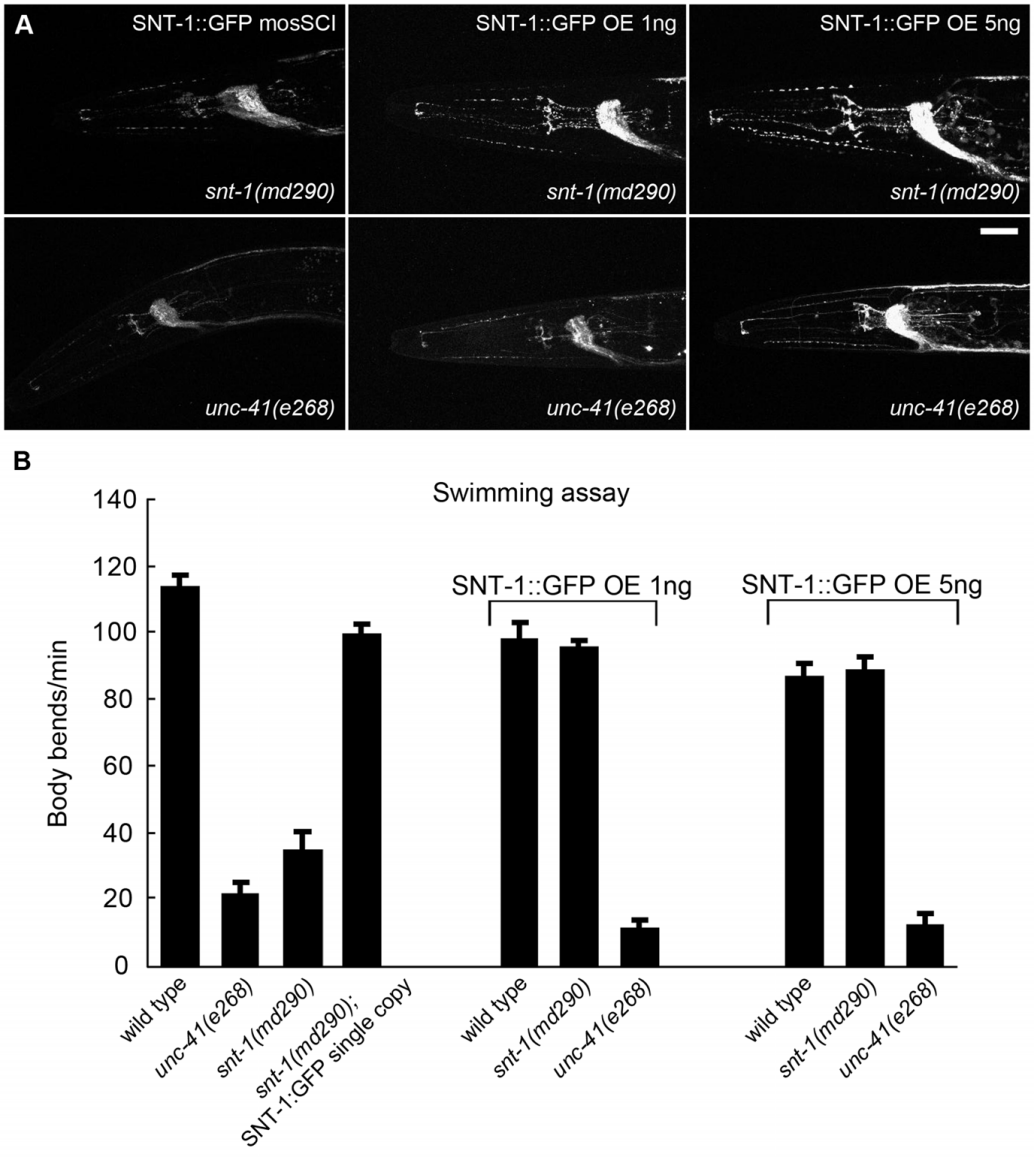
Overexpression of synaptotagmin does not rescue *unc-41* mutants. (A) Images of synaptotagmin (SNT-1::GFP) under different overexpression conditions. All images were taken at identical settings. High levels of tagged synaptotagmin were observed in the nerve rings of *snt-1(md290)* or *unc-41(e268)* mutants when injected at 5 ng/µl. Scale bar is 2 µm. (B) Swimming assays. Details are described in Materials and Methods. Error bars indicate SEM. Rescue of *unc-41* mutant phenotypes was not observed with the *snt-1::GFP* construct injected at 1 or 5 ng/µl.

If stonin’s primary function is to recruit synaptotagmin to sites of endocytosis, then providing excess synaptotagmin might restore enough synaptotagmin to synaptic vesicles to compensate for the lack of stonin. Overexpression of synaptotagmin in *Drosophila* is reported to rescue the lethality and synaptic vesicle recycling defects associated with *stonedB* mutations ([20], but note [37]). In *C. elegans*, we used several different SNT-1 overexpression approaches, but there was no rescue of *unc-41* mutant phenotypes. When the *snt-1::GFP* construct was expressed at endogenous levels by single copy insertion [38], or overexpressed from an extrachromosomal array (*snt-1::GFP* construct injected at 1 and 5 ng/µl), the tagged protein was localized to synapses (Figure 8A) and fully rescued the *snt-1* null phenotype (Figure 8B). When injected at 25 ng/µl, synaptotagmin overexpression was evident both by imaging and phenotype: SNT-1::GFP accumulated at the surface of neuron cell bodies (Figure S5A) and exhibited dominant-negative effects on the swimming rate of wild-type animals. These extra-chromosomal arrays were crossed into strains containing the *unc-41* alleles *e268* or *md134* (5.9 kb deletion, Figure 1A) to evaluate suppression. In these *unc-41* mutants, SNT-1::GFP diffused into axonal regions (Figure 8A), and the animals did not exhibit rescue even with very high expression levels (Figures 8B, S5B, S5C). We also overexpressed GFP-tagged or untagged SNT-1 from several different promoters and did not observe rescue of *unc-41* phenotypes (see Materials and Methods).

### Endocytosis Deficits in *unc-41* Mutants

**Figure 9:**
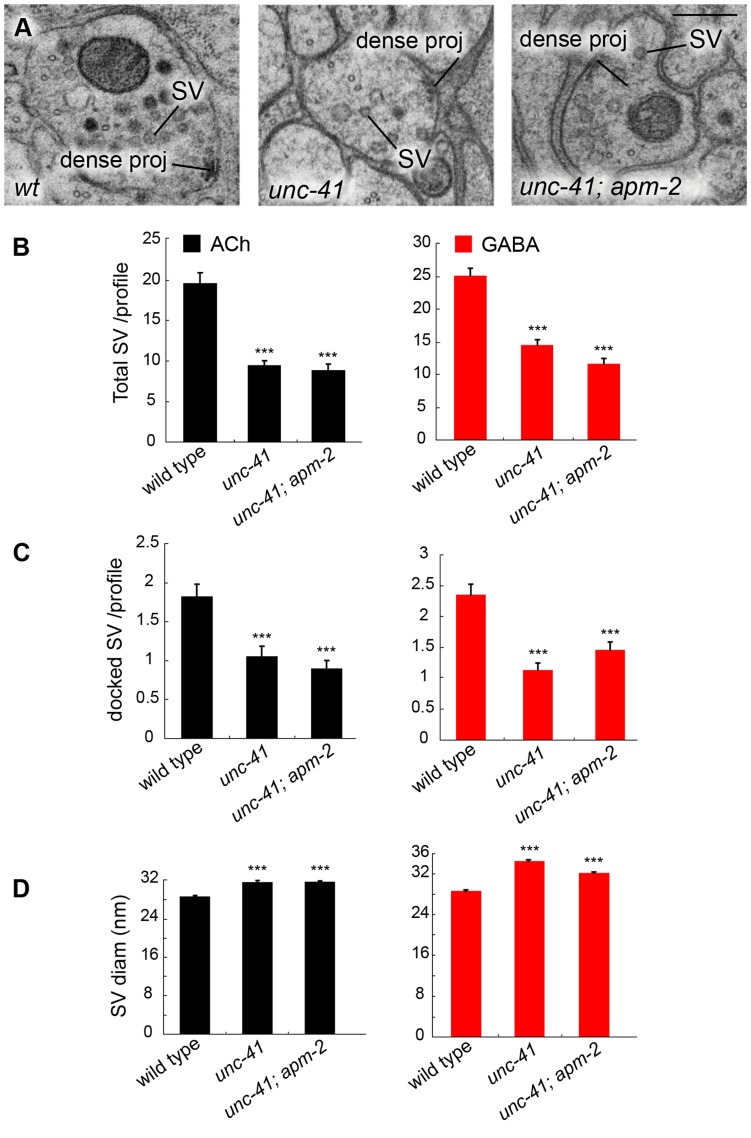
*unc-41* mutants have fewer synaptic vesicles at synapses. (A) Representative images of neuromuscular junctions in ventral nerve cords of the wild type, *unc-41(e268)*, and *unc-41(e268); apm-2(e840)* strains. Scale bar is 200 nm. SV, synaptic vesicle. (B) The total number of synaptic vesicles is reduced in *unc-41* and *unc-41; apm-2* mutants. (C) The number of docked synaptic vesicles is reduced in *unc-41* and *unc-41; apm-2* mutants. (D) Vesicle diameters are slightly increased in *unc-41* and *unc-41; apm-2* mutants. All data were obtained exclusively from profiles containing a dense projection. Values in panels B, C, and D represent means; error bars indicate SEM. The number of synapses scored in Panels B and C for wild type, *unc-41*, and *unc-41; apm-2* were 38, 40, and 30, respectively for ACh synapses, and 36, 45, and 39, respectively for GABA synapses. The number of synaptic vesicles measured for the data in panel D for wild type, *unc-41*, and *unc-41; apm-2* were 733, 380, and 265, respectively for ACh synapses, and 904, 652, and 453, respectively for GABA synapses. Triple asterisks denote significant difference from wild type (P<0.001).

To determine if synaptic vesicle endocytosis is affected in *unc-41* mutants, we quantified synaptic vesicles at neuromuscular junctions using electron microscopy (Figure 9A). We observed a 50% reduction of vesicles in both ACh and GABA motor neurons (Figure 9B). The number of docked vesicles was reduced proportionally (Figure 9C), suggesting that the phenotype was not caused by a specific deficit in docking. In addition, there was a modest but significant increase in the diameter of synaptic vesicles from 28.5 nm in wild type to ∼32 nm in *unc-41(e268)* mutants (Figure 9D), suggesting that UNC-41 regulates the size of synaptic vesicles.

Both stonins and µ2 adaptins contain µHDs, and the reduction in synaptic vesicles in *unc-41* mutants is comparable to that observed in *apm-2* mutants; *apm-2* encodes the *C. elegans* µ2 subunit [32]. In both cases, the phenotype is less severe than the strong endocytosis defects observed in endocytosis mutants such as the synaptojanin mutant *unc-26* [39]. We considered the possibility that µ2 adaptin and UNC-41 provide redundant functions. To test this, we performed an ultrastructural analysis on the *unc-41(e268) apm-2(e840)* double mutant (Figure 9A). The ultrastructural phenotype of the double mutant was almost identical to that of the *unc-41* single mutant (Figures 9B–D). These data suggest that despite their shared structural domain, µ2 adaptin and UNC-41/stonin are not functionally redundant, but rather act at different steps in the same pathway during synaptic vesicle endocytosis.

## Discussion

In this study, we show that the *unc-41* gene encodes the stonin ortholog in *C. elegans*. The two isoforms of UNC-41 are expressed in neurons and are localized to synapses. Based on our analysis of null mutants, we suggest that UNC-41 performs at least two functions during synaptic vesicle endocytosis: recruitment of synaptotagmin and recruitment of membrane. Recruitment of synaptotagmin is likely due to a direct interaction between UNC-41 and synaptotagmin; however, this interaction is not necessary for the localization of UNC-41. Recruitment of membrane is likely mediated by interactions between UNC-41 and AP2.

### Stonin Protein Structure and Functional Conservation

Members of the stonin family share specific structural features, but also exhibit considerable sequence diversity. The length of the protein varies among species and ranges from ∼900 amino acids in many mammals to 2099 amino acids in the trematode *Schistosoma mansoni*. The C-terminal stonin- and μ-homology domains (which together comprise ∼450–490 amino acids) are well-conserved, but the N-terminal one-half to three-quarters of the different proteins have very little sequence similarity.

However, despite such variability, the N-terminal regions of stonin proteins share common features which suggest mechanistic and functional similarities. These include proline-rich regions which bind SH3 domains of proteins such as endophilin and intersectin, NPF motifs which bind the EH domains of proteins such as eps15 and intersectin [10], and DPF, FxDxF, and WxxF motifs, which bind the “ear” domains of the α subunit of AP2 [10], [28]–[30]. In this regard, perhaps the two most divergent family members are *C. elegans* UNC-41 (containing WxxF, FxDxF, and DPF ear-binding motifs but no EF-hand-binding motifs), and *Drosophila melanogaster* STNB (containing seven NPF EF-hand-binding motifs but no ear-binding motifs). However, despite the apparent differences in protein interaction capabilities, we found that expression of a *stonedB* cDNA rescued *unc-41* mutant phenotypes and transgenic STNB protein was properly trafficked to synapses (Figure 6). Thus, although STNB and UNC-41 may employ distinct molecular mechanisms, STNB can nevertheless find its way to the right place and perform its proper function(s) in an otherwise all-worm cellular environment.

An unusual feature of UNC-41/stonin proteins from nematodes is the C-terminal Type 2 (YxY) PDZ-binding motif [31]. This motif was present in UNC-41 homologs from all nematode species that were analyzed, including several *Caenorhabditis* species, *Ascaris suum*, *Brugia malayi*, *Loa loa*, *Meloidogyne hapla*, and *Pristionchus pacificus*. The significance of this motif and its presumed interaction with a PDZ-domain containing synaptic protein is not clear. Once again, the rescue of *unc-41* mutant phenotypes by transgenic expression of STNB suggests that, whatever function the PDZ-binding motif may perform, there are alternate mechanisms able to perform the same or comparable functions.

### *unc-41* Mutants

The present study describes the first unambiguous knockout of a stonin in any organism. Although there are mutations that eliminate the STNB protein in *Drosophila*, all of these mutations also eliminate or affect STNA, because the two proteins are encoded by a bicistronic message [18]. A complicating factor is that STNA is also involved in synaptic function [12], [40], perhaps in a manner antagonistic to STNB [41]. A description of the phenotypes of stonin 2 mutations in the mouse has not been published.

Some phenotypes of *unc-41* mutants have been reported previously, and include uncoordinated locomotion, reduced pharyngeal pumping, resistance to cholinesterase inhibitors, slow growth, small adult size, and elevated ACh levels [23], [24], [26]. These studies were performed using the canonical *e268* allele, and we have now shown that this allele confers the same (presumably null) phenotypes as the other non-transposon-associated alleles.

### Synaptotagmin Recruitment

Previous studies have shown that stonins are adaptor proteins that recruit synaptotagmin to endocytic sites. In *Drosophila stoned* mutants, synaptic synaptotagmin is reduced and the remaining synaptotagmin is mislocalized to axons [12], [18]. Although mouse knockout mutants have not been reported, stonin 2 promotes synaptotagmin endocytosis in mammalian cells as well. Expression of human stonin 2 in cultured fibroblasts or neurons promotes the endocytosis of surface synaptotagmin 1 [16], and endocytosis requires an interaction domain on stonin 2 [19]. Our analysis of *unc-41* mutants supports a role for UNC-41 in localization of synaptotagmin to presynaptic terminals in *C. elegans*: counts of visible puncta in null *unc-41* mutants indicates that SNT-1::GFP localization to many synapses is below the detection level of what is a ‘synapse’ to a blinded scorer (Figures 4, S2, S3).

In contrast, we find that synaptotagmin is not required for UNC-41 recruitment. We show that UNC-41 is properly localized in SNT-1 mutants (Figure 5) and mutants deficient in other synaptotagmins (Figure S4), suggesting that binding to synaptotagmin is not necessary for the synaptic localization of stonin proteins. Furthermore, we have shown that proper localization of UNC-41 does not require any synaptic vesicle components (Figure 5B). These data strongly support a unidirectional relationship between stonins and synaptotagmin. Although previous experiments indicated that mutation of a putative synaptotagmin binding site (KYE>AAA) disrupted localization of stonin to synapses [19], it is likely that this mutation also disrupts the proper folding and trafficking of stonin, or binding to proteins other than synaptotagmin.

### Does UNC-41 Perform Additional Synaptic Functions?

Although it has been clear that stonins function as sorting adaptors for synaptotagmin, it has not been clear whether they have additional targets or functions. We addressed this issue in two ways: we analyzed the phenotypes of *snt-1 unc-41* double mutants, and we evaluated the effects of synaptotagmin overexpression.

If the only function of UNC-41 is to recruit synaptotagmin, then we would expect that the phenotypes of *snt-1 unc-41* double mutants would be comparable to those of the single mutants. However, we observed that the *snt-1 unc-41* double mutants were significantly more impaired than the *snt-1* mutant by itself (Figure 7); these data suggest that UNC-41 has additional functions beyond the recruitment and retrieval of synaptotagmin.

Next, if the primary function of UNC-41 is to recruit synaptotagmin to sites of endocytosis, then providing excess synaptotagmin might restore enough synaptotagmin to synaptic vesicles to compensate for the lack of a stonin. In *Drosophila*, overexpression of synaptotagmin is reported to rescue the lethality and synaptic vesicle recycling defects associated with *stonedB* mutations ([20], but note [37]). However, although we tried several different SNT-1 overexpression paradigms, we observed no rescue of *unc-41* mutant phenotypes in *C. elegans* (Figures 8B, S5B, S5C).

Taken together, these data suggest the existence of additional UNC-41 functions beyond the recruitment of synaptotagmin at synapses. A possible caveat to this interpretation is that there are multiple synaptotagmin genes in *C. elegans*, and UNC-41 may act as endocytic adaptors for one or more of these proteins. In addition, these experiments do not identify specific functions beyond recruiting synaptotagmin. One possibility is that UNC-41 mediates the retrieval of other synaptic vesicle proteins. In *Drosophila*, the levels of cysteine string protein and the vesicular glutamate transporter (VGluT) were also reduced in *stoned* mutants [34], [40]. However, we only observed modest mislocalization of other synaptic vesicle proteins, including synaptobrevin, synaptogyrin, VGAT, and VAChT, in *unc-41* mutants (Figures 4, S2, S3). Thus, if other vesicle proteins are specifically targeted for recycling by UNC-41, their identities are not currently known.

### Membrane Recruitment

Contradictory data obscure the role of stonins in membrane endocytosis. In *Drosophila stonedB* hypomorphs, vesicles are clustered and docked normally at active zones of third instar larvae [34]. However, in embryonic lethal *stoned* mutants, there is a 50% reduction in the number of synaptic vesicles at synapses, and large diameter vesicles were observed in synaptic profiles [12]. *C. elegans unc-41* mutants also exhibit a 50% reduction in synaptic vesicle numbers and vesicles are slightly larger in diameter, consistent with a defect in endocytosis of synaptic vesicle membrane [4], [42], [43].

The endocytic phenotype of *unc-41* mutants is similar to that of *apm-2* mutants [32]. Because both UNC-41 and µ2 adaptin contain µHDs, it was possible that these proteins act in a partially redundant manner. If this were true, the double mutant would exhibit a more severe reduction in synaptic vesicle number. However, the *unc-41 apm-2* double mutants exhibit the same ∼50% reduction of vesicles as *unc-41* or *apm-2* single mutants. These data suggest that stonin and µ2 act in the same endocytic pathway, and their µHDs are not functionally equivalent. In addition, *apm-2* mutants do not exhibit defects in synaptotagmin localization [32]. These data are consistent with experiments in flies and mammalian cultured cells demonstrating that the stonin and µ2 µHDs are not interchangeable [16], [34].

The lack of additive effects in *unc-41 apm-2* double mutants is surprising. We expected that impairing a major adaptor complex in addition to UNC-41 would worsen the endocytosis deficit. However, ∼50% of the vesicles and the majority of vesicle cargo do not depend on either of these adaptors, suggesting that either they are not essential for retrieval, or another adaptor (or adaptor complex) can function redundantly. We have recently examined *apa-2* mutants (deficient for α adaptin); *apa-2* mutants exhibit only a small reduction in the number of synaptic vesicles. By contrast, *apa-2 apm-2* double mutants exhibit a severe decrease in synaptic vesicles. These data suggest that α adaptin acts in parallel to µ2; a manuscript describing these results is in preparation.

In summary, our data suggest that UNC-41/stonin in *C. elegans* functions in two processes in synaptic vesicle endocytosis. It localizes synaptotagmin to synapses, possibly by recruiting synaptotagmin to sites of synaptic vesicle endocytosis. In addition, UNC-41/stonin functions with the µ2 subunit of the AP2 complex to recover membrane during endocytosis. Because synaptotagmin is required for membrane endocytosis [42], [44], [45], it is likely that synaptotagmin retrieval and membrane retrieval are part of a single pathway. Specifically, UNC-41 could recruit synaptotagmin, synaptotagmin could activate µ2-AP2, and AP2 would initiate membrane endocytosis. Alternatively, µ2, stonin, and synaptotagmin may function as part of a complex that regulates membrane recruitment. Each of these proteins probably has additional functions as well; for example, µ2 is also required for stabilization of the AP2 complex. Eliminating any one of these proteins is likely to have less severe consequences than eliminating multiple components. These two models provide intriguing directions for future research.

## Materials and Methods

### Strains and Mutants

The wild-type strain is Bristol N2. Additional strains are in List S1. The *unc-41* alleles with *e* allele designations were isolated at MRC, Cambridge, and were generously provided by Jonathan Hodgkin: *e252*, *e268*, and *e650* were isolated by Sydney Brenner after EMS mutagenesis, *e850* was isolated by Padmanabhan Babu after P^32^ decay mutagenesis, and *e1162* and *e1294* were isolated by Don Riddle after ICR191 mutagenesis. The *unc-41* alleles with *md* allele designations were isolated in the Rand laboratory as spontaneous mutants resistant to the acetylcholinesterase inhibitor aldicarb [24], [26]. They were identified as *unc-41* alleles by genetic mapping and complementation tests, and were outcrossed at least six times. The *unc-41* alleles *n2163* (EMS) and *n2913* (diepoxybutane) were isolated by Erik Jorgensen and Bob Horvitz, and *ox63* (ENU) was isolated by Erik Jorgensen. The *snt-2(tm1711)III*, *snt-3(tm2426)V, and snt-6(tm3686)II* alleles were obtained from Shohei Mitani (Tokyo Women’s Medical College, Tokyo), and the *snt-4(ok503)I* allele was provided by the *C. elegans* Gene Knockout Consortium.

### Molecular Biology, Sequence Analysis, and Cloning of *unc-41*

Standard molecular biology techniques were used for preparing *C. elegans* DNA and RNA, screening cDNA and genomic libraries, and performing Northern blot analyses. The *unc-41* gene was cloned by transposon tagging [46], using the Tc1 insertion allele *md1175*. The genomic phage RM#231L was isolated from a *C. elegans* genomic library prepared by Heidi Browning and Tom Blumenthal. It contains the complete *unc-41* gene with 3529 bp of upstream sequence. The genomic sequence was determined by “primer walking,” using plasmid subclones from phage RM#231L as templates. The genomic subclones were also used to screen two cDNA libraries prepared by Bob Barstead. Genomic clones and cDNAs were completely sequenced on both strands using the *fmol*® DNA Cycle Sequencing System (Promega, Madison, WI). The *unc-41* gene, which has the cosmid designation C27H6.1, was subsequently sequenced by the *C. elegans* Genome Sequencing Consortium (Genbank Accession number NM073165), with identical results. We also note that this gene was transiently known as *apt-10* [47].

### Analysis of Mutations

Mutations were initially analyzed by Southern blot or PCR analysis. Some of the mutations were associated with altered fragment lengths, which allowed us to determine the approximate nature of the rearrangement. Further analysis involved amplification of specific 1–2 kb *unc-41* genomic regions using direct single-worm PCR. The precise deletion endpoints or insertion sites were determined by sequencing purified PCR products. PCR products from those mutants without rearrangements were used for Restriction Endonuclease Fingerprinting analysis [48]. Several of the mutations were analyzed by direct sequencing of PCR-amplified genomic DNA. Most of the sequencing was performed by the DNA Sequencing Center at Oklahoma State University.

### Plasmids

Expression plasmids for transformation utilized the pPD49.26 vector or derivatives (gifts of Andy Fire, Stanford School of Medicine). Promoter regions from the *unc-41* gene were cloned into the first multiple cloning site of pPD49.26. A CFP, GFP, or YFP reporter gene carrying a nuclear localization signal (gift of Andy Fire, Stanford School of Medicine) was cloned into the second multiple cloning site [49]. The *unc-41* cDNA clone yk40f7 was obtained from Yuji Kohara (National Institute of Genetics, Mishima, Japan), and the *stonedB* cDNA was generously provided by Mani Ramaswami (Trinity College, Dublin, Ireland). The GFP::UNC-41B (pMG13) construct from Jung *et al.* [19] was used to examine the localization of UNC-41 in synaptotagmin mutants.

### Antibodies

An UNC-41 fusion protein was expressed in *E. coli* and used to generate polyclonal antibodies. The plasmid RM#198p contains coding sequences corresponding to residues 1124 to 1693 of the UNC-41A protein in the *malE* expression vector pIH902 (New England Biolabs). The resulting fusion protein was insoluble and was purified as inclusion bodies. The fusion protein was injected into two goats (gt216 and gt220). Polyclonal antibodies were affinity purified against the UNC-41 fusion protein as described previously [50]. Specificity of the antibody preparation was demonstrated by the absence of immunostaining in *unc-41(e268)* mutants (Figure 3A).

Additional antibodies used in this study include anti-UNC-17/VAChT([MAb1403) [51], anti-SNB-1/VAMP (Ab1092) [52], anti-SNT-1/synaptotagmin (R558) [27], and anti-UNC-10/RIM (Ab271) [53].

### Immunofluorescence Staining

Nematodes were stained using a modified freeze-fracture procedure as previously described [54]. For multiple-labeling experiments, animals were fixed in 1% formaldehyde in phosphate buffered saline (PBS) for 30 min on ice. For single-labeling experiments with anti-UNC-17 Abs, animals were fixed in methanol followed by acetone.

### Microinjection

DNA transformation methods for *C. elegans* were essentially as described by Mello et al. [55]. The final DNA concentration of each injection mix was 100 ng/µl. This target concentration was obtained with the addition of GeneRuler™ 1 kb DNA Ladder (#SM0311) or pBluescript™. pMG13 was injected at 1 ng/µl in all UNC-41::GFP experiments. The coinjection marker *Punc-122::GFP* was injected at 50 ng/µl.

### Synaptotagmin Overexpression

A *snt-1::GFP* construct (pRH353) was injected at 1, 5, or 25 ng/µl into wild-type animals. The coinjection marker *Punc-122::GFP* was injected at 50 ng/µl. The same extrachromosomal arrays were subsequently crossed into *snt-1(md290), unc-41(e268)*, and *unc-41(md134)* backgrounds. Rescue of the *unc-41* phenotype was not observed in these synaptotagmin overexpression experiments. In addition, expression of a SNT-1::GFP fusion under control of the pan-neural synaptobrevin (*snb-1*) promoter failed to rescue the *unc-41* phenotype. Because it was remotely possible that the GFP tag interfered with SNT-1 function, we also overexpressed untagged SNT-1. A *snt-1* genomic plasmid (pRH264) was injected into *unc-41(e268)* at 25 ng/µl along with the coinjection marker *Prab-3::GFP* at 75 ng/µl. Rescue of *unc-41* phenotypes was not observed in these transgenic animals either (data not shown).

### Microscopy and Imaging

Confocal images of UNC-41 and YFP::STNB expression patterns were collected on a Leica TCS NT confocal microscope using 40× or 63× magnification objectives, at 512×512 or 1024×1024 pixels. Images were cropped to size, assembled, and annotated using Adobe® Photoshop® CS2. All images within a given experiment were collected in the same session using identical settings and were processed identically.

For all other images, worms were immobilized by using 2% phenoxy propanol and imaged on a Pascal LSM5 confocal microscope using Zeiss plan-Neofluar 10×0.3 NA or Zeiss plan-apochromat 63×1.4 NA oil objectives.

Quantification of puncta in sublateral nerve cords: Young adult hermaphrodites were immobilized with 2% phenoxypropanol and imaged using a Pascal LSM5 confocal microscope with a Zeiss plan-apochromat 63×1.4 NA oil objective. Ten 100 µm regions of sublateral nerve cords (approximately midway between the level of the retrovesicular ganglion) were imaged using identical settings. The experimenter was blinded to the genotype of the animal and the identity of the fluorescent protein, and the total number of fluorescent puncta was determined for each 100 µm region.

### Electron Microscopy

Wild type (N2), *unc-41(e268)*, and *unc-41(e268); apm-2(e840)* adults were prepared in parallel for transmission electron microscopy. All animals were raised at room temperature (22.5°C). After high-pressure freezing, chemical fixation and substitution, and embedding, mutant and control blocks were blinded, and ribbons of 250 contiguous ultrathin (33 nm) serial sections were collected from two animals of each genotype using an Ultracut 6 microtome (Leica). Images were collected on a Hitachi H-7100 125keV electron microscope using a Gatan slow scan digital camera. Image analysis was performed using ImageJ software (v1.38 NIH). Axonal processes in the ventral nerve cord were reconstructed for the VA and VB ACh, and VD GABA motor neurons. Neuromuscular synapses were identified by the presence of a varicosity containing synaptic vesicles surrounding a dense projection oriented toward the muscle. The total number of neuromuscular junctions analyzed was 19 synapses (ten ACh and nine GABA) for *unc-41* and 18 synapses (nine ACh and nine GABA) for *unc-41 apm-2* double mutants. Vesicles were analyzed only in sections containing a dense projection. The numbers of synaptic vesicles (∼30 nm), dense-core vesicles (∼40 nm), and large vesicles (>40 nm) at each synapse were counted, and their distances from presynaptic specialization and plasma membrane were determined, as well as their diameters. The average number of synaptic vesicles and docked vesicles per profile were calculated for each set of images containing any part of the presynaptic dense projection. The numbers for each profile were averaged to obtain the final value.

### Behavioral Assays

All behavioral measurements were performed at ∼22°C on young adult hermaphrodites. To measure swimming, individual worms were transferred to M9 buffer and allowed to adapt to the liquid environment for 60–90 s. The number of body bends was then counted for 60 or 120 s for each genotype (n ≥7). For expulsion measurements, ten young adults of each strain were observed for 10–12 consecutive defecation cycles, and the success rate was determined as the percentage of cycles that culminated in an enteric muscle contraction (EMC).

### Statistical Tests

The two tail unpaired Student’s *t*-test was used for statistical analyses.

## Supporting Information

Figure S1 **Map of** ***unc-41*** **mutations.** Molecular details of each mutation are in Table S1. Tc1 insertions were identified at five distinct sites; they are labeled A - E in the direction of *unc-41* transcription.
(TIF)

Figure S2 **The localization of SNT-1::GFP is disrupted in the body-sublateral nerve cords of an** ***unc-41*** **mutant homozygous for the deletion allele** ***md134*** **(see Figure S1 and Table S1).** Scale bar is 10 µm.
(TIF)

Figure S3 Above**:** Localization of synaptic vesicle proteins in GABA neurons in *unc-41* mutants. Synaptic localization of SNT-1::GFP is disrupted in *unc-41* mutants; fluorescence is visible in a commissure (arrowhead), although some fluorescence is also observed at puncta along the dorsal nerve cord. Minor mislocalization to axons is observed for other proteins in *unc-41* mutants. Images are of dorsal nerve cords. Below: Localization of endocytosis proteins in *unc-41* mutants. GFP-tagged α adaptin (APA-2::GFP) and tdTomato tagged endophilin (UNC-57::tdTomato) are not severely disrupted in the sublateral neurons of *unc-41(md230)* mutants. Scale bars are 10 µm.
(TIF)

Figure S4 **GFP::UNC-41 is properly localized to synapses in different synaptotagmin (** ***snt*** **) triple mutants.** GFP::UNC-41B is localized to synapses in *snt-4(ok503); snt-1(md290); snt-2(tm1711)* and *snt-6(tm3686) snt-1(md290); snt-3(tm2426)* triple mutants. Scale bar is 20 µm.
(TIF)

Figure S5 **Dominant negative effects are observed when synaptotagmin is highly overexpressed.** (A) Representative images of animals expressing a *snt-1::GFP* construct injected at 25 ng/µl. All images were taken at identical settings. The scale bar is 20 µm. (B) Nomarski images of a *snt-1(md290)* mutant, a *snt-1(md290)* mutant carrying a *snt-1::GFP* overexpressing array, an *unc-41(e268)* mutant, and an *unc-41(e268)* mutant carrying a *snt-1::GFP* overexpressing array. The scale bar is 100 µm. (C) Synaptotagmin overexpression does not rescue the *unc-41* phenotype. The extent of rescue was quantified with swimming assays (body bends/min). Error bars indicate SEM. All *t*-tests are compared with the swimming rate of wild-type animals without the SNT-1 transgene. Triple asterisks denote significant difference from wild type (P<0.001).
(TIF)

List S1 **Supplementary strain Information.**
(DOCX)

Table S1 **Molecular basis of** ***unc-41*** **mutations.** Alleles are listed alphabetically. Exon and amino acid numbering are for the UNC-41A protein. DNA sequences correspond to the direction of transcription. Tc1 insertions were identified at five distinct sites (labeled A - E in the direction of *unc-41* transcription; see Figure S1). The orientation of the Tc1 insertion (<< or >>) represents the orientation of the transposon open reading frames.
(DOCX)
