# Supplementary figures and images for: UNC-41/Stonin Functions with AP2 to Recycle Synaptic Vesicles in Caenorhabditis elegans
Source: PLoS One. 2012 Jul 10;7(7):e40095. doi: 10.1371/journal.pone.0040095 (PMC3393740; doi:10.1371/journal.pone.0040095)

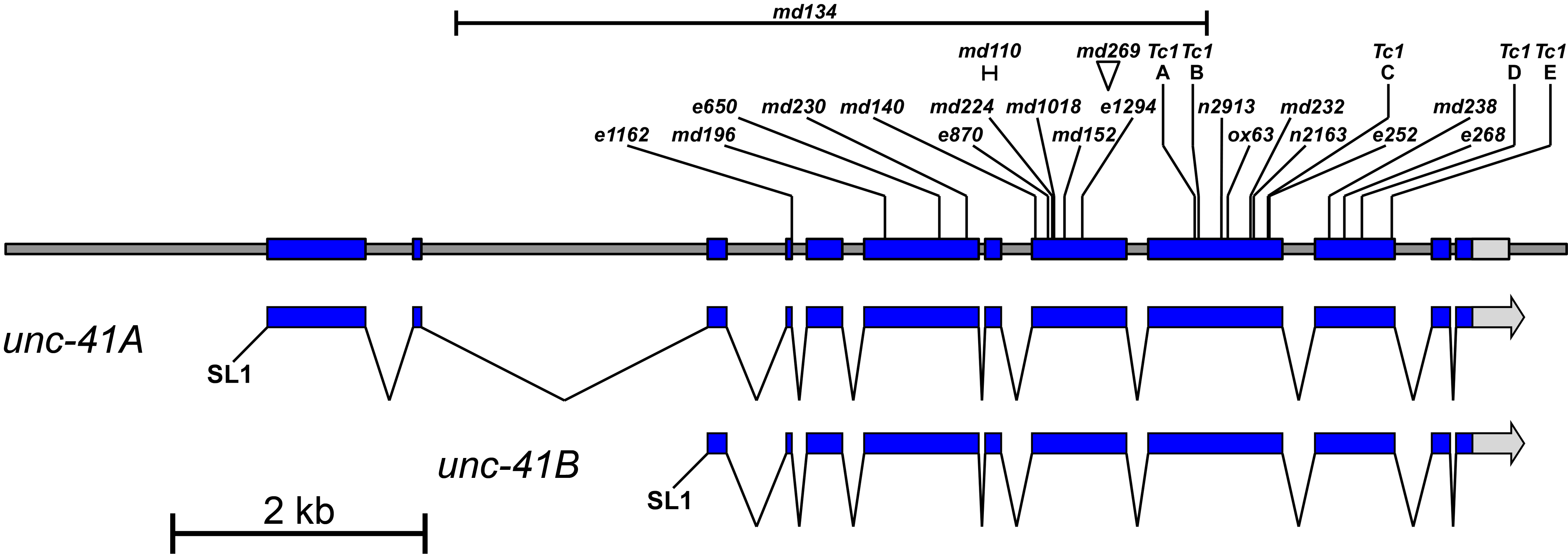

Supplement: Figure S1 — Map of unc-41 mutations. Molecular details of each mutation are in Table S1. Tc1 insertions were identified at five distinct sites; they are labeled A - E in the direction of unc-41 transcription. (TIF) [file pone.0040095.s001.tif]

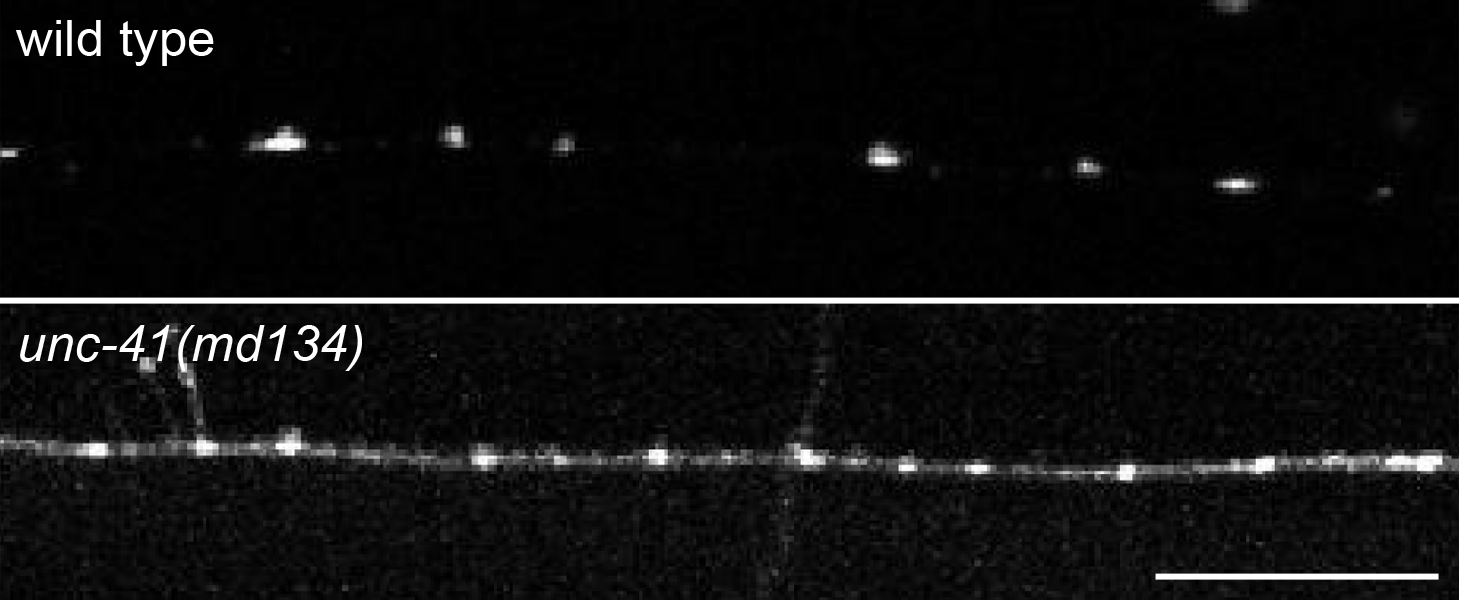

Supplement: Figure S2 — The localization of SNT-1::GFP is disrupted in the body-sublateral nerve cords of an unc-41 mutant homozygous for the deletion allele md134 (see Figure S1 and Table S1). Scale bar is 10 µm. (TIF) [file pone.0040095.s002.tif]

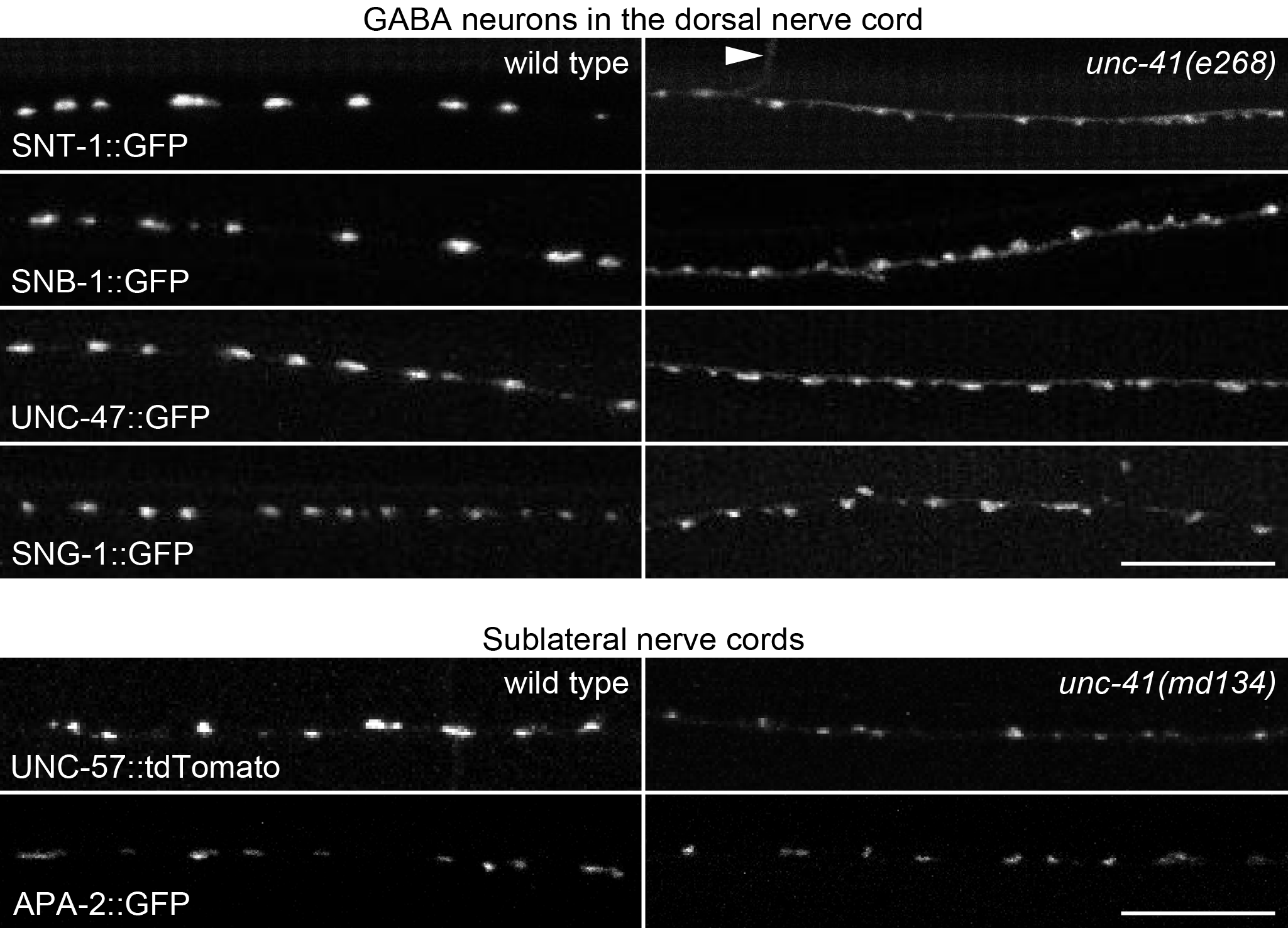

Supplement: Figure S3 — Above: Localization of synaptic vesicle proteins in GABA neurons in unc-41 mutants. Synaptic localization of SNT-1::GFP is disrupted in unc-41 mutants; fluorescence is visible in a commissure (arrowhead), although some fluorescence is also observed at puncta along the dorsal nerve cord. Minor mislocalization to axons is observed for other proteins in unc-41 mutants. Images are of dorsal nerve cords. Below: Localization of endocytosis proteins in unc-41 mutants. GFP-tagged α adaptin (APA-2::GFP) and tdTomato tagged endophilin (UNC-57::tdTomato) are not severely disrupted in the sublateral neurons of unc-41(md230) mutants. Scale bars are 10 µm. (TIF) [file pone.0040095.s003.tif]

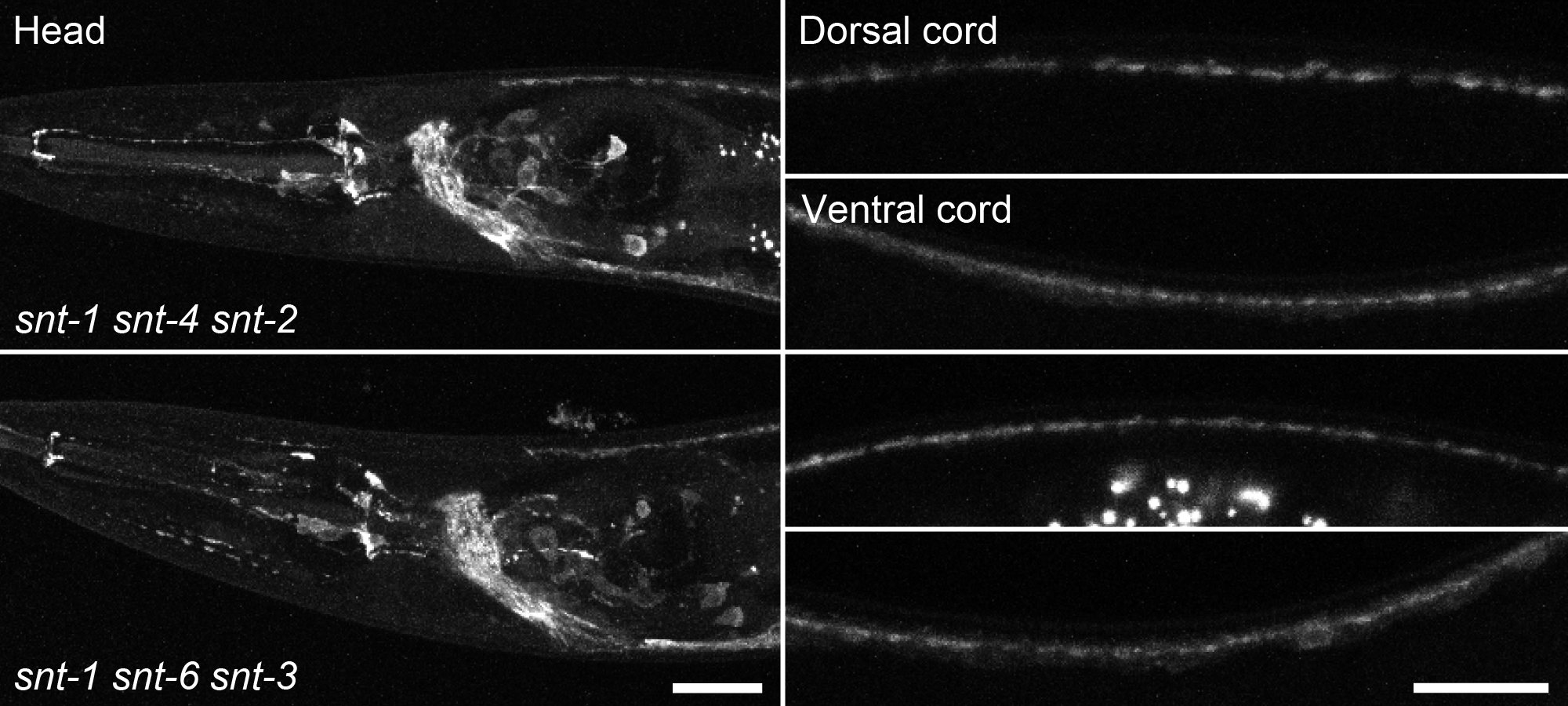

Supplement: Figure S4 — GFP::UNC-41 is properly localized to synapses in different synaptotagmin ( snt ) triple mutants. GFP::UNC-41B is localized to synapses in snt-4(ok503); snt-1(md290); snt-2(tm1711) and snt-6(tm3686) snt-1(md290); snt-3(tm2426) triple mutants. Scale bar is 20 µm. (TIF) [file pone.0040095.s004.tif]

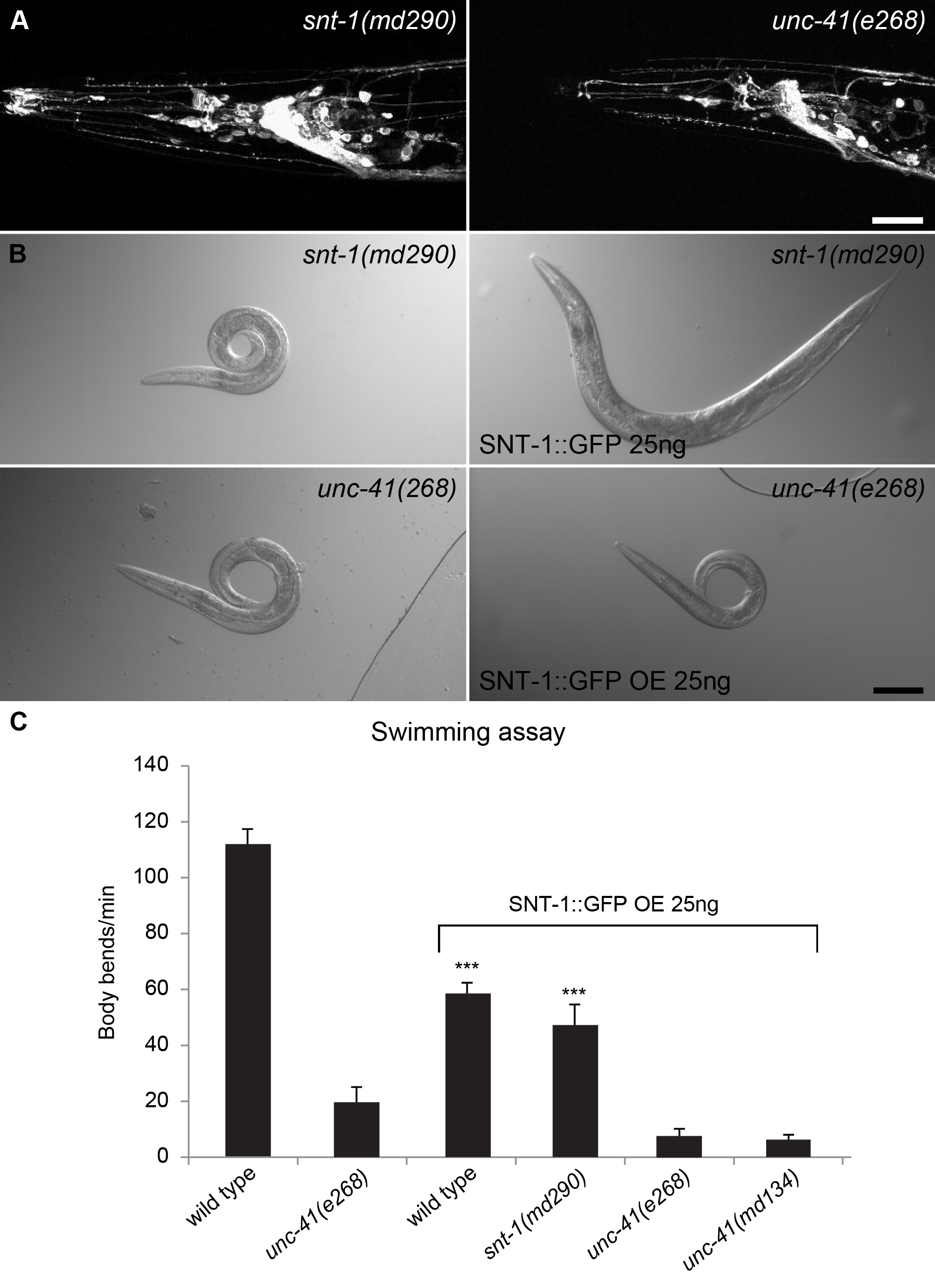

Supplement: Figure S5 — Dominant negative effects are observed when synaptotagmin is highly overexpressed. (A) Representative images of animals expressing a snt-1::GFP construct injected at 25 ng/µl. All images were taken at identical settings. The scale bar is 20 µm. (B) Nomarski images of a snt-1(md290) mutant, a snt-1(md290) mutant carrying a snt-1::GFP overexpressing array, an unc-41(e268) mutant, and an unc-41(e268) mutant carrying a snt-1::GFP overexpressing array. The scale bar is 100 µm. (C) Synaptotagmin overexpression does not rescue the unc-41 phenotype. The extent of rescue was quantified with swimming assays (body bends/min). Error bars indicate SEM. All t-tests are compared with the swimming rate of wild-type animals without the SNT-1 transgene. Triple asterisks denote significant difference from wild type (P<0.001). (TIF) [file pone.0040095.s005.tif]
