# Supplementary material for: UNC-41/Stonin Functions with AP2 to Recycle Synaptic Vesicles in Caenorhabditis elegans
Source: PLoS One. 2012 Jul 10;7(7):e40095. doi: 10.1371/journal.pone.0040095 (PMC3393740; doi:10.1371/journal.pone.0040095)
Supplement: List S1 — Supplementary strain Information. (DOCX) [file pone.0040095.s006.docx]

**List S1 - Supplementary Strains**

All *oxSi* strains were generated by mosSCI [1], so that exogenous genes were inserted as single copies.

The reference strains for *unc-41(e268)V* are EG1531 and CB268.

The reference strains for *snt-1(md290)II* are EG1026 and RM1613.

The *snt-1; unc-41* double mutants used were:

MT7918: *snt-1(md184)II; unc-41(e268)V*

RM3286: *snt-1(md290)II; unc-41(e268)V*

RM3657: *snt-1(md290)II; unc-41(md152)V*

RM1677: *unc-41(md152)V* (control)

RM1610: *snt-1(md184)II* (control)

The strain used to determine the *unc-41* expression pattern was RM3280: *pha-1(e2123)III; mdIs157[Punc-41A::NLS-CFP]; mdEx762[Punc-41B::NLS-YFP pBX]*.

The strains used for GFP::UNC-41 localization experiment were:

EG4775: *snt-1(md290)II; oxEx1072[Punc-41A::GFP::unc-41B Punc-122::GFP]*

EG4741: *oxEx1050[Punc-41A::GFP::unc-41B Punc-122::GFP]*

EG6426: *snt-4(ok503)I; snt-1(md290)II; snt-2(tm1711)III; oxEx1528[Punc-41A::GFP::unc-41B Punc-122::GFP]*

EG6427: *snt-6(tm3686) snt-1(md290)II; snt-3(tm2426)V; oxEx1529[Punc-41A::GFP::unc-41B Punc-122::GFP]*

The strain used for UNC-104 trafficking assay was RM977: *unc-104(e1265)II.*

The strains used to assess rescue by STNB were:
RM2086: *pha-1(e2123)III; mdEx186[pBX]*  (pBX rescue of *pha-1*)

RM2655: *pha-1(e2123)III; unc-41(e268)V; mdEx251[pBX]* (pBX rescue of *unc-41; pha-1*)

RM2683: *pha-1(e2123)III; unc-41(e268)V; mdEx279[Punc-41A::stnB pBX]*

RM3644: *pha-1(e2123)III; unc-41(e268)V; mdEx1062[Punc-41A::YFP::stnB pBX]*

RM3582: *snt-1(md290)II; pha-1(e2123)III; mdEx1062[Punc-41A::YFP::stnB pBX]*

The double mutant strain used for electron microscopy was EG4216: *unc-41(e268)V; apm-2(e840)X.*

The strains used for localization of synaptic proteins were:
EG5882: *snt-1(md290)II; oxSi114[snt-1::GFP unc-119(+)]IV*
EG6428: *oxSi114[snt-1::GFP unc-119(+)]IV; unc-41(e268)V*
NM1233: *jsIs219[sng-1::GFP rol-6]II*
EG6556: *jsIs219[sng-1::GFP rol-6]; unc-41(e268)V*
EG6503: *oxSi168[EGFP::snb-1 unc-119(+)]I; unc-119(ed3)III; snb-1(js124)V*
EG6555: *oxSi168[EGFP::snb-1 unc-119(+)]I; unc-41(e268)V*EG6579: *unc-119(ed3)III; oxSi402[Punc-47::snt-1::GFP unc-119(+)]IV*
EG6609: *oxSi402[Punc-47::snt-1::GFP unc-119(+)]IV; unc-41(e268)V*EG5717: *unc-119(ed3)III; oxSi36[unc-47::GFP unc-119(+)]IV*
EG6429: *oxSi36[unc-47::GFP unc-119(+)]IV; unc-41(e268)V*
MT8247: *lin-15(n765ts) nIs52[Punc-25::snb-1::GFP lin-15(+)]X*
EG3049: *unc-41(e268)V; lin-15(n765ts) nIs52[Punc-25::snb::GFP lin-15(+)]X*
EG6163: *unc-119(ed3)III; oxSi184[Punc-47::sng-1::GFP unc-119(+)]IV*
EG6557: *oxSi184[Punc-47::sng-1::GFP unc-119(+)]IV; unc-41(e268)V.*

EG7320: *oxSi199[unc-57::tdTomato cbunc-119(+)]II*

EG7173: *oxSi199[unc-57::tdTomato cbunc-119(+)]II; unc-41(md230)V*

EG7321: *oxSi253[Prab-3::apa-2::GFP cbunc-119(+)]II*

EG7322: *oxSi253[Prab-3::apa-2::GFP cbunc-119(+)]II; unc-41(md230)V*

The strains used for SNT-1 overexpression experiments were:
RM1676: *unc-41(md134)V*
EG6558: *oxEx1549[snt-1::GFP(1ng) Punc-122::GFP]*
EG6559: *snt-1(md290)II; oxEx1549[snt-1::GFP(1ng) Punc-122::GFP]*
EG6560: *unc-41(e268)V; oxEx1549[snt-1::GFP(1ng) Punc-122::GFP]*
EG6561: *unc-41(md134)V; oxEx1549[snt-1::GFP(1ng) Punc-122::GFP]*
EG6562: *oxEx1548[snt-1::GFP(5ng) Punc-122::GFP]*EG6563: *snt-1(md290)II; oxEx1548[snt-1::GFP(5ng) Punc-122::GFP]*
EG6564: *unc-41(e268)V; oxEx1548[snt-1::GFP(5ng) Punc-122::GFP]*
EG6565: *unc-41(md134)V; oxEx1548[snt-1::GFP(5ng) Punc-122::GFP]*
EG6430: *oxEx1534[snt-1::GFP(25ng) Punc-122::GFP]*EG6431: *snt-1(md290)II; oxEx1534[snt-1::GFP(25ng) Punc-122::GFP]*
EG6432: *unc-41(e268)V; oxEx1534[snt-1::GFP(25ng) Punc-122::GFP]*
RM3413: *pha-1(e2123)III; unc-41(e268)V; mdEx939[Psnb-1::snt-1B::GFP(2.5ng) pBX]*
RM3414: *pha-1(e2123)III; unc-41(e268)V; mdEx940[Psnb-1::snt-1A::GFP(2.5ng) pBX]*

**REFERENCE**

1. Frøkjær-Jensen C, Davis MW, Hopkins CE, Newman BJ, Thummel JM et al. (2008) Single-copy insertion of transgenes in *Caenorhabditis elegans*. Nat Genet 40: 1375-1383.
