# Supplementary material for: UNC-41/Stonin Functions with AP2 to Recycle Synaptic Vesicles in Caenorhabditis elegans
Source: PLoS One. 2012 Jul 10;7(7):e40095. doi: 10.1371/journal.pone.0040095 (PMC3393740; doi:10.1371/journal.pone.0040095)
Supplement: Table S1 — Molecular basis of unc-41 mutations. Alleles are listed alphabetically. Exon and amino acid numbering are for the UNC-41A protein. DNA sequences correspond to the direction of transcription. Tc1 insertions were identified at five distinct sites (labeled A - E in the direction of unc-41 transcription; see Figure S1). The orientation of the Tc1 insertion (<< or >>) represents the orientation of the transposon open reading frames. (DOCX) [file pone.0040095.s007.docx]

| **Allele** | **Molecular Summary** | **Flanking Sequences** |
| --- | --- | --- |
| *e252* | Base substitution; W1357>Stop in exon 9 | GCCAATGTATACGGAAAGATG **/ G>A /** ATTCGATTTGAAGCTTTGGA |
| *e268* | Base substitution; W1469>Stop in exon 10 | TTCCGAGAAGAACGTCATTG **/ G>A /** GGAGTAGGATCAATTCATTC |
| *e650* | Base substitution; Q640>Stop in exon 6 | ATCAAGGCCAGGAATATCAA **/ C>T /** AAGAATATTATCAAAACCAC |
| *e870* | C>AATGAATG in exon 8 | ACAAGGATTTGTTAATGAAT **/ C>AATGAATG /** GACACAAAAAGAGATTCAAA |
| *e1162* | 1-bp insertion in exon 4 | CAGATGCACCACACCAAGGG **/ G /** GTAAGTAAGCTCATTTCTTT |
| *e1294* | 1-bp insertion in exon 8 | TCAGCATCTGCGATTCCCCC **/ C /** AAGAAAGAGAAGTTCAATAA |
| *md110* | 100-bp deletion; removes part of exon 7 | TACATAATTATAATACAATT **/ /** AAGAAGATGCATGGGCGCAG |
| *md134* | 5950-bp deletion; removes exons 3-8 and part of exon 9; new junction sequence is TCTCCAG, putative in-frame splice site | CAATTAGACATCACTTTCTC **/ /** CAGATACAACGCTTCAAGCA |
| *md140* | 2-bp deletion in exon 8 | CTGCAGTAAAATCAACAGAG **/ /** TACTCTGAAAAACTTGGAGG |
| *md152* | 1-bp deletion in exon 8 | ATGAAGAAGACGCAATGGAT **/ /** GGGCTGCACAAGAGTTAGCG |
| *md196* | 17-bp insertion in exon 6 | ACATGCTGGATATGA **/ GCTGGATATGAATATGA /** TCAAGGAGATTTTTA |
| *md224* | AG>CAC in exon 8 | GATTCAAAGGTTGACAGAAG **/ AG>CAC /** AAGAAAATGGAGAAACTTCA |
| *md230* | Base substitution; E712>STOP in exon 6 | AAGAGTTATCTCCAACACCA **/ G>T /** AAGCTTCATCATCTACTGGA |
| *md232* | 1-bp deletion in exon 9 | ATTTGTAGACAAGGAAGGTA **/ /** TCTGTCAGATCAGAAAGCCC |
| *md238* | 19-bp deletion in exon 10 | CGAAATTAGAATTGAAGCAA **/ /** ACGTACACGTGGATCCGATG |
| *md269* | 176-bp tandem duplication in exon 8 | AAATCCAAAAGGAGATCATG **/ /** AGATCTAGTTTTGTAGCAGA |
| *md1018* | 2-bp deletion in exon 8;  also A>C substitution in exon 8 | AGAAGAGAAGAAAATGGAGA **/ /** CTTCAGAAGAAAAAGCTAAA;  GAAGAAAAAGCTAAAGCAGC **/ A>C /** AGGAAAGAAAGCTGCAACCC |
| *md1087* | Tc1 insertion in exon 9 (site B) | CATGGACTGACACTGTATTT **/ Tc1>> /** ATACAATGACAAAAAAGATG |
| *md1097* | Tc1 insertion in exon 9 (site A) | AAGATGTTGGAAACCTTGTT **/ <<Tc1 /** ATGTTCGACTCCATGGACTG |
| *md1105* | Tc1 insertion in exon 10 (site D) | GGCTAAATATGAACATGTAT **/ Tc1>> /** ATCGTTCACTTGTATGGAGG |
| *md1132* | Tc1 insertion in exon 10 (site E) | CGTGGAAAAGTTTGTCAGAT **/ <<Tc1 /** ATGTTGCTAAATATCAGTAT |
| *md1138* | Tc1 insertion in exon 10 (site D) | GGCTAAATATGAACATGTAT **/ Tc1>> /** ATCGTTCACTTGTATGGAGG |
| *md1140* | Tc1 insertion in exon 10 (site D) | GGCTAAATATGAACATGTAT **/ <<Tc1 /** ATCGTTCACTTGTATGGAGG |
| *md1148* | Tc1 insertion in exon 10 (site D) | GGCTAAATATGAACATGTAT **/ Tc1>> /** ATCGTTCACTTGTATGGAGG |
| *md1159* | Tc1 insertion in exon 10 (site E) | CGTGGAAAAGTTTGTCAGAT **/ Tc1>> /** ATGTTGCTAAATATCAGTAT |
| *md1160* | Tc1 insertion in exon 10 (site D) | GGCTAAATATGAACATGTAT **/ <<Tc1 /** ATCGTTCACTTGTATGGAGG |
| *md1170* | Tc1 insertion in exon 10 (site E) | CGTGGAAAAGTTTGTCAGAT **/ <<Tc1 /** ATGTTGCTAAATATCAGTAT |
| *md1175* | Tc1 insertion in exon 10 (site D) | GGCTAAATATGAACATGTAT **/ Tc1>> /** ATCGTTCACTTGTATGGAGG |
| *md1200* | Tc1 insertion in exon 9 (site C) | AAGATATTCTGCCAATGTAT **/ <<Tc1 /** ACGGAAAGATGGATTCGATT |
| *md1250* | Tc1 insertion in exon 10 (site D) | GGCTAAATATGAACATGTAT **/ Tc1>> /** ATCGTTCACTTGTATGGAGG |
| *n2163* | Base substitution; R1316>STOP in exon 9 | CAGATCAGAAAGCCCGCGTT **/ C>T /** GACTCTTTTGTCTTGCATTC |
| *n2913* | 13-bp deletion in exon 9;  also 1-bp insertion in exon 9 | CGTCCAGGACAAATCAGTCG **/ /** CACATCACAAAATATGGTTT;  TTGTAGACAAGGAAGGTATT **/ T /** CTGTCAGATCAGAAAGCCCG |
| *ox63* | Base substitution; C1247>STOP in exon 9 | CTAGAACATTCTGCTCAATG **/ T>A /** ACAGTTCTCTTAAAGTTCGG |

**Table S1.** Molecular basis of *unc-41* mutations. Alleles are listed alphabetically. Exon and amino acid numbering are for the UNC-41A protein. DNA sequences correspond to the direction of transcription. Tc1 insertions were identified at five distinct sites (labeled A - E in the direction of *unc-41* transcription; see Figure S1). The orientation of the Tc1 insertion (<< or >>) represents the orientation of the transposon open reading frames.
